# Partisan Divergence in Fertility Change Before and During the COVID-19 Pandemic in Florida

**DOI:** 10.1007/s11113-025-09972-0

**Published:** 2025-09-15

**Authors:** Heather M. Rackin, Christina M. Gibson-Davis, Courtney E. Williams, Dustin Hughes, Seunghwan Yoo

**Affiliations:** 1 Department of Sociology, Louisiana State University, 125 Stubbs Hall, Baton Rouge, LA 70803, USA; 2 Sanford School of Public Policy, Duke University, 228 Rubenstein Hall, Box 90312, Durham, NC 27708, USA; 3 Population Research Center, The University of Texas at Austin, 305 E. 23rd Street, Stop G1800 RLP 2.602, Austin, TX 78712-1699, USA; 4 Department of Sociology, Western Washington University, Arntzen Hall 511, 516 High Street, Bellingham, WA 98225, USA; 5 Department of Sociology, Louisiana State University, 122 Stubbs Hall, Baton Rouge, LA 70803, USA

**Keywords:** Fertility, Voting, COVID-19 pandemic, Political identity, Polarization

## Abstract

Motivated by political-based differences in pandemic perceptions, this study analyzed whether Republican- and Democratic-leaning counties exhibited differential fertility shifts, leading to a partisan fertility gap. As COVID-19 emerged, the political right dismissed the threat of the virus, while the political left emphasized it as a major crisis. These contrasting views may have led to diverging fertility responses between those living in Democratic- and Republican-leaning areas. Using county-level data from Florida, difference-in-difference models predicted quarterly change in fertility rates between 2018 and 2022. Models estimated the partisan fertility gap (e.g., Republican-Democratic difference in fertility rate changes relative to before the pandemic) as a function of 2020 Trump vote share. The partisan fertility gap widened during the pandemic’s early months, as fertility in Republican-leaning counties declined less than in Democratic-leaning counties. This gap was only observed for White women and was robust to controlling on time-varying potential confounders (unemployment rate and racial composition changes). The partisan gap was short-lived, however. Results suggest that politically-charged contexts where would-be-parents lived may have affected pandemic-induced fertility shocks and demonstrates the need to understand fertility changes in the context of the broader political environment—a vital endeavor given record-low fertility and unprecedented political polarization in the United States.

## Introduction

With the onset of the COVID-19 pandemic U.S. birth rates, mirroring trends seen in other Western countries, declined precipitously. In January of 2021 (reflecting conceptions that occurred early in the pandemic), the total fertility rate was 1.6, an 8.5% decrease relative to January of 2020 (Bailey et al., 2023). This reduction was in line with general expectations that crises, characterized by heightened uncertainty and anxiety about the future, often precipitate fertility delays or declines (Chabé-Ferret & Gobbi, 2018; Comolli & Vignoli, 2021; Guetto et al., 2020; Hofmann & Hohmeyer, 2013; Schneider, 2015). After reaching a nadir in January of 2021, birth rates have increased, with some evidence suggesting that the latter months of the pandemic ushered in a “baby boom” that will reverse a decades-long decline in birth rates (Bailey et al., 2023). Despite these recent increases, little doubt exists that the onset of the COVID-19 pandemic corresponded with a large negative shock to the nation’s fertility rate.

In this study, we argue that pandemic-induced fertility shocks echo, at least in part, the politically-charged contexts in which potential parents resided in. As COVID-19 emerged, the political right downplayed the virus’s threat and questioned its reality (Kates et al., 2020), while the political left emphasized the pandemic as a large-scale crisis that highlighted perceived government failures (Lopez, 2021; Pew, 2020). These differing narratives likely led to vastly divergent pandemic experiences. While Democratic-leaning areas were embedded in a context of fear with vastly altered daily activities, Republican-leaning areas exhibited less dread and saw fewer changes. For instance, people in Republican-leaning areas physically distanced less, felt more comfortable returning to pre-pandemic behaviors, and expressed greater optimism about the future; conversely, people in Democratic-leaning areas altered their behavior more to align closely with public health measures and reported greater worry about the future (Allcott et al., 2020; Burns, 2021; Gadarian et al., 2021; Hartig, 2022; Hsiehchen et al., 2020; Young et al., 2022).

These varying pandemic experiences, shaped by the political context, may have led to divergent fertility responses. In politically right-leaning contexts, influenced by Republican messaging that downplayed the COVID-19 pandemic, people may have maintained or even increased their pre-pandemic fertility as government financial assistance and more women at home reduced the economic and opportunity costs of a birth, making it easier to enact their preferred traditional family roles. In contrast, people in politically left-leaning areas, exposed to alarming messages and seeing vast behavioral changes, may have had more difficulty enacting their preferred egalitarian gender roles and felt more fear about the future, and thus been less likely to have a baby. Given this, a partisan fertility gap may have emerged early in the pandemic, where those in Democratic-leaning areas abstained from childbearing but those in Republican-leaning areas continued to bear children.

As the COVID-19 pandemic endured, partisan fertility gaps likely shrank with Democratic-leaning area’s fertility increasing to meet or exceed Republican-leaning areas. Those in Democratic-leaning locales would likely have more births (or stop postponing) because of rising employment and wealth (Aladangady et al., 2023; Falk et al., 2021) along with tempered anxiety (as the pandemic shock wore off) and more optimism about the future (with Biden’s 2020 presidential election win and successful vaccine development).

Work on the association between partisan context and fertility responses to events is scant, but political leanings are important for fertility-related domains. The most relevant studies found that the 2016 presidential election of Republican Donald Trump predicted an increase in fertility in Republican voting counties (Dahl et al., 2022) and that the strength of the association between changes in fertility and unemployment in the Great Recession varied by a state’s partisan lean (more Democratic voting states had weaker associations; Morgan et al., 2011). Despite the paucity of studies on partisan context and fertility responses to events, individual political identity is predictive of fertility desires and intimate behaviors, including whom to marry or befriend (Gimpel & Hui, 2015; Huber & Malhotra, 2017; Iyengar et al., 2018; Rackin & Gibson-Davis, 2024), and macro-political context is related to family and fertility (Campisi et al., 2023; Lesthaeghe & Neidert, 2017). Broadly, social context is associated with fertility theoretically and empirically (Fiori et al., 2014; Ghimire, 2017; Johnson-Hanks et al., 2011; Karabchuk et al., 2022; Vignoli et al., 2020).

To grasp if differing pandemic partisan contexts led to changes in fertility behavior, we analyze whether Republican- and Democratic-leaning counties showed differential fertility shifts in response to COVID-19. Data come from county-level voting and quarterly birth records in the state of Florida. As the third most populous state with a mix of Republican and Democratic counties, Florida is an ideal context to study partisan responses to the pandemic. Using time-series methods with county and time fixed effects, we compare excess fertility rates (excess fertility is defined as changes in the general fertility rate for a given quarter in a given year relative to the same quarter’s average in prior years [2014–2017]). Thus, excess fertility adjusts for seasonality and year-to-year fluctuations in births in Republican- and Democratic-leaning counties (defined relative to the share of votes cast for Trump in the 2020 presidential election). The partisan lean of counties was measured in three ways: (1) whether Trump received a majority (≥ 50%), (2) whether Trump received a *super*majority of votes (≥ 60%), and (3) a continuous percentage measure of Trump vote. We model the excess fertility rate as a function of a county’s level of partisanship to obtain estimates of the difference in Republican–Democratic excess fertility rate (hereafter referred to as the “partisan fertility gap”) for birth quarters occurring between 2018 and 2022 relative to the 2014–2017 average. With these difference-in-difference models, we show changes in the partisan fertility gap, relative to 2014–2017 averages, for three periods: the pre-pandemic period (January 2018–December 2019), the pandemic births period (January–September 2020), and the pandemic conceptions period (October 2020–June 2022). Models also control for changes in county-level racial/ethnic composition and unemployment rate, two time-varying factors that could confound the partisanship-fertility link.

We also analyzed whether 2020 Trump support had differential effects on White versus non-White women’s fertility rates. Insofar as most non-White women identify as, and are close to, Democrats (Pew, 2018), county-level 2020 Trump vote share may be less informative about their own or their close associates’ politically-informed views. This holds true even if non-White parents-to-be reside in largely Republican-leaning areas, as they themselves and those in their social circles are likely Democrats. White women, though, are relatively evenly split between the parties (Pew, 2021); as such, county Trump vote provides more insight into the views they or those with whom they closely interact hold (i.e., a White woman living in a heavily Republican- [Democratic-] leaning county is likely to identify with, or be closely tied to people in, the predominate political group). If this intuition is correct, then county-level 2020 Trump vote share should be more predictive of White, rather than non-White, women’s fertility.

This is the first study that leverages the extremely divergent interpretations of the pandemic by Republicans and Democrats to explore if partisan contexts influenced childbearing, one of the most profound decisions a person can make. In so doing, our work speaks to broader questions about the salience of the political, and social, context for family behaviors.

## Background

### Pandemic Fertility Changes

The onset of the COVID-19 pandemic coincided with a decrease in births (Bailey et al., 2023). The decline in fertility was relatively short-lived, however. After reaching a nadir in January of 2021 (reflecting births conceived in the early months of the pandemic), births began to rebound and surpassed pre-pandemic rates. Indeed, more than a decades’ long trend of declining fertility reversed in the latter half of 2021 and into 2022 (Bailey et al., 2023).

Changes in fertility during the pandemic likely resulted from a variety of factors. Some of these factors may have decreased fertility, such as increased unemployment, worsening physical and mental health, changes in immigration, and decreased physical contact (Nobles et al., 2024). Other factors may have led to increases in fertility: decreased access to medical care may have led to shifts in contraceptive practices, for example. Economic conditions also improved for some, through enhanced unemployment assistance and government stimulus payments.

Fertility behavior was also likely responsive to pandemic anxiety, with anxiety level shaped by the community and social context. During the first pandemic months, fertility intentions declined, with declines predicted by anxiety about the future (Guetto et al., 2020; Manning et al., 2022). Further, controlling on COVID-19 cases, fertility rebounds were smaller in states with masking mandates, suggesting that pandemic worries and community context mattered (Kearney & Levine, 2023). When making fertility decisions and faced with uncertainty attendant to crises, people look for cues from those around them and trusted media to interpret how concerned they should be and what it means for their own and potential children’s future (Vignoli et al., 2022).

Evidence as to why fertility trends rebounded is still emerging, but the cognitive-social model of fertility decision making may help explain this reversal. Theoretically, fertility choices, as indicated by the cognitive-social model, are governed by material conditions operating at multiple levels (e.g., the adequacy of an individual’s material resources to raise a child, relationship status, community resources amenable to childrearing) but also by cognitive and affective understandings of the world (Johnson-Hanks et al., 2011). Cognitive and affective notions of a situation allow people to determine what is occurring and how to respond. Material and cognitive factors do not act independently of each other, but rather interact to influence fertility decision making (Johnson-Hanks et al., 2011). For instance, material conditions favorable to childrearing may only increase fertility if people feel and believe that having a(nother) child in their current circumstances and anticipated future would be a good choice (or at least not a bad one). Thus, the cognitive-social model would provide expectations that material conditions during the pandemic (e.g., higher government financial support, more flexibility to work from home) would result in higher fertility, but not until people felt less cognitive and emotional discomfort about the pandemic.

Below, we first detail partisan differences in beliefs and behaviors during the pandemic. We next describe how political context might have shaped fertility decisions, potentially leading to partisan fertility gaps early in the pandemic.

### Pandemic Partisanship

In the United States, intense political polarization has led to vast partisan divergence in media narratives and thought leaders’ interpretations of the pandemic (Fan et al., 2023; Gadarian et al., 2021; Gollust et al., 2020; Iyengar et al., 2012; Layman et al., 2006). The Republican party and politically-right media consistently downplayed the severity of COVID-19 and stated on multiple occasions that it was nothing to worry about and/or that it was just like the flu (Bunker, 2020; Kates et al., 2020). Thought leaders on the political right, such as President Trump, stated innumerable times that COVID-19 was going to “disappear” (Kates et al., 2020; Wolfe & Dale, 2020). Contrastingly, Democratic thought leaders, such as Dr. Anthony Fauci (then Director of the U.S. National Institute of Allergy and Infectious Diseases and later chief medical advisor to President Biden), consistently stated that the pandemic threat was substantial (Brennan, 2020; Feuer, 2020). Democrats and politically left-leaning media reported the COVID-19 pandemic as a large-scale crisis where the government continued to “fail” (Lopez, 2021). Democratic-leaning media outlets in the United States suggested that the pandemic highlighted the problematic nature of the nation’s safety net, healthcare systems, gender relations, and the government’s ability to respond to families’ and individuals’ needs (Diamond, 2021; Menon, 2021; Qato, 2020).

Given these politically-divergent portrayals of the COVID-19 pandemic, it is unsurprising that Republicans and Democrats had different cognitive appraisals of and behavioral responses to the crisis. Cognitively, Republicans were more likely to believe that people were overreacting to COVID-19 (Green & Tyson, 2020) whereas Democrats were more likely to view the pandemic as a threat (Schaeffer, 2021). By mid-2020, Republicans, relative to Democrats, were more optimistic about the future and economy (Burns, 2021; Hartig, 2022; Pew, 2020), and felt more comfortable returning to pre-pandemic behaviors (e.g., parties; Gollwitzer et al., 2020; Pew, 2020). Republicans were also less likely than Democrats to express worry about the future across multiple domains such as ability to return to normal, school closures, and getting sick (Gadarian et al., 2021). Behaviorally, Republicans, as compared to Democrats, were less likely to increase handwashing, avoid crowds, or curtail travel (Allcott et al., 2020; Gadarian et al., 2021). These individual behaviors were also present at the aggregate level as data show less physical distancing in Republican-versus Democratic-leaning areas (Allcott et al., 2020; Hsiehchen et al., 2020). Taken together, these hyper-partisan narratives likely had a strong influence on COVID-era perceptions and behavioral responses.

### Political Partisanship and Fertility

Political partisanship likely shaped cognitive and affective understandings during the COVID-19 pandemic, as limited evidence indicates that partisan context informs interpretations of a situation’s amenability to childbearing. During the Great Recession, unemployment was less predictive of fertility declines in Democratic-leaning states than in Republican-leaning states, suggesting President Obama’s 2008 election engendered optimism about the recession and its effects (Morgan et al., 2011). After President Trump’s 2016 election, fertility rates increased more in Republican-leaning counties than in Democratic-leaning ones, possibly due to differing shifts in optimism about the current and future situation (Dahl et al., 2022). Outside of the United States, radical-right-wing party support in Swedish municipalities was linked to a lower probability of having a child, perhaps because this support reflected declining certainty and generalized trust—key factors in childbearing decisions (Comolli & Andersson, 2021).

The above findings are consistent with the cognitive-social model and narrative framework, which detail that fertility choices are driven by cognitive notions of childbearing, particularly around optimism for the future, and salient social identities. These notions are molded by messages and behaviors observed within one’s social milieu, such as social networks and media (Johnson-Hanks et al., 2011). Partisanship might be key as it is an increasingly relevant marker of identity (Mason, 2018) and correlate of fertility desires (Rackin & Gibson-Davis, 2024) that shapes social interactions (Huber & Malhotra, 2017) and optimism (Bonaparte et al., 2017).

Given the divergent partisan views of the COVID-19 pandemic, we suggest that an important factor in determining U.S. fertility responses is likely partisanship insofar as partisan context informs interpretation of events as to whether a given situation is amenable to childbearing. Early in the pandemic the different interpretations of the scope and reality of the crisis and concomitant behavioral changes likely led to diverging fertility. Potential parents in Republican-leaning areas, likely believing the pandemic was a non-event, might not have altered their childbearing plans or may have even increased their fertility, whereas would-be-parents living in Democraticleaning areas were surrounded by grave messages about the pandemic’s deep and profound impacts, which may have fueled anxiety about the future and prompted reduced fertility.

The cognitive-social model argues that material conditions interact with cognitive meanings to produce behavior, and during the pandemic, potential parents in Republican- and Democratic-leaning areas likely interpreted similar constraints differently. Women faced higher unemployment rates than men due to their overrepresentation in service-oriented jobs (Albanesi & Kim, 2021), but job losses, combined with government stimulus and favorable unemployment benefits, may have allowed families to enact traditional family roles without significant income loss. Republicans, more likely to idealize male-breadwinner family models (Burge, 2024), may have viewed this as a favorable opportunity for childbearing, especially since couples enacting traditional family roles likely felt less disrupted by school and daycare closures. In contrast, Democrats, who tend to support more gender egalitarian views that emphasize the importance of both partners working (Burge, 2024), may have refrained from having children despite government support due to job insecurity for either partner. Given Democrats’ more egalitarian family views, they may have seen potential employment and schooling disruptions as obstacles to childbearing. Democratic partisans’ anxieties may have also been exacerbated by the Republican-led government’s initial COVID-19 response. Thus, for would-be parents in Democratic-leaning areas, the pandemic’s abrupt onset likely drove fertility declines similar to those seen in prior crises, such as the Great Recession (Cherlin et al., 2013). We hypothesize that these differing interpretations and responses created a partisan fertility gap, with fertility declining less in Republican-leaning counties than in Democratic-leaning ones, leading to a positive Republican-Democratic gap in fertility change for births conceived early in the pandemic.

As the COVID-19 pandemic continued to unfold, though, partisan gaps may have attenuated. Democratic areas may have increased their fertility, as the shock and uncertainty perhaps gave way to more hopeful messages circulating in Democratic social milieus. Fertility in Democratic-leaning areas may also have increased. Contexts became less hostile to childbearing and career as unemployment declined (Falk et al., 2021) and stimulus monies provided greater resources (Aladangady et al., 2023). When combined with tempered anxiety (as the pandemic became a “new normal”) and more optimism about the future (given the 2020 Democratic presidential win and effective vaccine development), residents in Democratic-leaning counties may have been more willing to have children.

It is also likely that, in the two years before the COVID-19 pandemic, partisan fertility gaps were small or non-existent. Small or null pre-pandemic gaps would likely occur because there were no large crises in which partisans exhibited vastly different behavioral and attitudinal responses.

Here we use the divergent changes in beliefs and behaviors by partisan context that occurred early in the COVID-19 pandemic to understand how changes in views of the world and future and behaviors in potential parents’ social milieu may have been associated with the socially and individually transformative event of bringing new children into the world. This work highlights the importance of considering partisanship as a key contextual factor in shaping fertility behaviors and broader family dynamics.

### Variation by Racial Group

If partisan contexts affect pandemic fertility change because they reflect potential mothers’ own beliefs or those of their relevant social others, then we would expect county-level vote share to predict fertility change but only among groups for whom the county-level Trump vote provides a good proxy for their politically-informed pandemic views. As we detail below, county-level 2020 Trump vote share is likely a poor proxy for the politically influenced pandemic views of non-White women and their close associates, but a better proxy for those of White women. Thus, if women’s and/or their close associates’ views matter for fertility change in the COVID-19 pandemic, then partisan divergence should be evident only for changes in White women’s fertility rates.^1^

County-level vote share is likely uninformative as to the partisan views for non-White women or the views non-White women were exposed to from close associates. Non-White women are highly unlikely to vote Republican; for example, 6% of Black women voted for Trump in 2020. Thus, regardless of the vote share for Trump in a county where a Black voter lived, Black women were highly unlikely to be Trump supporters themselves or be surrounded by close associates expressing politically right-leaning messages. As a result, county-level Trump support does not proxy non-White women’s politically-informed views or the messages they heard and internalized and, thus, should be unrelated to fertility rate changes among non-White women. Indeed, it is more plausible that non-White women’s fertility rates in both Trump supporting and non-Trump supporting counties align with patterns in Democratic-leaning counties because a large majority of non-White women and their close associates were Democrats.

For White women, however, county-level Trump vote may be a better proxy for the partisan influenced pandemic views they, or those with whom they closely interact, held. Unlike non-White women, White women vote in nearly equal shares for Republican and Democratic candidates. In the 2016 and 2020 presidential elections, the Trump vote share for White women was 47% and 53%, respectively (Pew, 2021). When this political evenness is combined with partisan geographical sorting (Tam Cho et al., 2013), most White women who lived in Trump voting counties likely either personally endorsed Trump’s messages or were at least exposed through their close associates. Conversely, most White women living in counties where Trump did not receive a majority of the vote likely did not endorse Trump’s messages and instead heard Democratic ones from close associates. Thus, we reason that county-level 2020 Trump support is a reasonable, though not perfect, proxy for White women’s own politically shaped views or of those with whom they closely interact. We therefore expect that county-level measures of 2020 Trump vote share would be predictive of White women’s pandemic fertility responses, whereas we would not expect 2020 Trump support to be predictive of non-White women’s fertility responses.

## Data and Methods

Data came from state vital statistics reports on the quarterly counts of all births and births by race (White and non-White; FloridaHealth, 2024).^2^ Births were observed quarterly^3^ between the first quarter of 2014 (January–March) to the second quarter of 2022 (April–June). Out of the 67 Florida counties, 56 were included in the analysis. We excluded 11 counties^4^ because they had at least one quarter with very few births (defined as less than the 10th percentile of births, or fewer than 22 births, 16 White births, or 4 non-White births).^5^

While recognizing that results are limited to one state, we nevertheless believe that Florida offers advantages for this study. To begin, Florida is a large state (third most populous), accounting for 6% of all U.S. births in 2020 (Census Bureau, 2020; March of Dimes, 2022). To put Florida’s population size in context, if it were a country, it would rank slightly below Australia and above Malawi. Second, demographically, Florida is broadly reflective of the U.S. population (Census Bureau, 2020). In Florida, 77% of residents are White and 17% are Black, relative to 76% and 14%, respectively, in the United States overall; Floridians are somewhat more likely to be Hispanic, 27% versus 19%, and Foreign-born, 21% versus 14%. The share of Floridians less than age 65 (79%) is also comparable to the national average (83%). Third, at the county level, Florida exhibits good variability, as a majority in 18% of its 67 counties voted for the Democratic candidate and a majority in 82% voted for the Republican candidate. Other U.S. states have too few counties to get meaningful variation (e.g., Delaware has three) and some states have no variation either because Trump failed to get a majority of the vote in any county (three states) or Trump got a majority in over 90% of counties (15 states). Fourth, using a single state allowed us to hold time-varying state-level factors constant. For instance, Republican Ron DeSantis became governor in 2019 and state response to the COVID-19 pandemic would affect all Florida counties. Finally, Florida vital statistics provides data very quickly; for instance, in this study we have data on births until June 2022, allowing us to observe conceptions when President Biden took office (at the time of writing, natality data for the United States were only available through 2021).

### Measures

The dependent variable was excess fertility, defined as how much fertility changed in a given quarter in our observation window (i.e., 2018–2022) compared to the same quarter’s 2014–2017 average. This operationalization allowed us to predict within-county changes in fertility rates relative to the 2014–2017 average found in the same season. As an example: Bay County, in the panhandle of Florida, had a fertility rate of 11.9 in the first quarter (January–March) of 2019. Over the 2014–2017 period, Bay County’s average fertility rate in the first quarter was 13.9. This county’s excess fertility rate was thus −2, as its fertility rate was two births per 1000 reproductive-age women lower in the first quarter of 2019 relative to its average first quarter fertility rate in 2014–2017. The excess fertility measure was preferred over other measures (such as the simple or log of fertility rate) as it shows within-county change but also takes the seasonality of fertility into account and smooths random year-to-year fluctuations by comparing observed fertility rates to the four-year fertility rate average before the COVID-19 pandemic (i.e., 2014–2017).

Our measure of excess fertility begins in the first quarter of 2018, as the prior four years (2014–2017) were used as a comparison period (see Online Supplement Table S1). The general fertility rate was calculated as the number of births in each quarter divided by the census’s mid-year estimate of the female population aged 15–50 years multiplied by 1000. For White women’s (non-White women’s) excess fertility, the denominator was the census’s mid-year estimates of White alone (non-White) female population aged 15–50 years (Florida vital statistics only provide White and non-White categories, and the vast majority of Hispanic births reflect those who are classified as White).^6^ We used mid-year population estimates in the conception year to be consistent with the measurement of unemployment.

To classify counties as either Republican- or Democratic-leaning, we used three measures of Trump vote share in the 2020 presidential election. All three measures are based on 2020 voting data from the MIT election lab (MIT, 2022). These measures were: (1) majority Trump support (≥ 50% vote share), (2) supermajority Trump support (≥ 60% vote share), and (3) a continuous measure of Trump vote share. All variables were measured at the county level. For the categorical measures, counties were classified as Republican-leaning (coded 1) if a majority (or supermajority) voted for Donald Trump, the Republican candidate, versus all others (coded 0).^7^ We used three measures because, together, they capture both the marginal effect of an increase in Trump support, as well as the effect of living in counties where votes were skewed or highly skewed towards Trump. We note that, even though the 2020 election occurred during our observation window, we preferred 2020 over 2016 voting data as it more accurately reflects partisan pandemic messaging (in robustness checks presented below, we discuss the similarity of results using 2016 Trump and 2018 gubernatorial data).

Time, included as a series of dichotomous indicators, refers to the quarter of birth. Eighteen birth quarters were included, from quarter 1 of 2018 (January–March) to quarter 2 of 2022 (April–June). The 18 birth quarters were divided into three periods to indicate the relative timing of the conception and birth vis-à-vis the pandemic (see Table 1): the pre-pandemic period, pandemic births period, and the pandemic conceptions period. The pre-pandemic period includes conceptions and births that both occurred before the pandemic (e.g., births realized between the first quarter of 2018 [January–March] and the last quarter of 2019 [October–December]).

The pandemic births period includes pre-pandemic conceptions but pandemic births (births that occurred during the first three quarters of 2020). We included this period because others have found fertility declined prior to the pandemic (Nobles et al., 2024) likely due to factors such as decreased immigration which declined a great deal in 2020 but rebounded in 2021 (Camarota & Zeigler, 2024). We note, though, that the first quarter of 2020 (January–March), contains primarily pre-pandemic births. The pandemic conceptions period includes quarters where both conceptions and births occurred during the pandemic (births from October–December 2020 to April–June 2022, with conceptions ranging from January–March 2020 to July–September 2021). The first quarter in this period contains a mixture of pre-pandemic and early pandemic conceptions (e.g., January–March 2020 conceptions). Importantly, the last year of observations in the pandemic conceptions period covers conceptions that occurred after Biden’s election.

As covariates, we included county-level measures of racial composition and unemployment, two factors that could change over time and differentially affect fertility in Republican- and Democratic-leaning counties. We included racial/ethnic composition in case certain subpopulations exhibited differential migration in response to the pandemic. For instance, immigration from outside of the United States was limited during the pandemic and fewer foreign-born Hispanics, who tend to have higher fertility and may move to Democratic-leaning counties, could have depressed fertility more in Democratic-leaning, rather than Republican-leaning, counties. We also controlled for changes in unemployment, insofar as tourism-related cutbacks experienced during the pandemic may have been concentrated in Democratic-leaning areas (e.g., Disney World in Orlando, Key West). Given negative correlations between unemployment rates and fertility (Behrman & Gonalons-Pons, 2020), lower pandemic fertility in Democratic-leaning counties could have stemmed from greater increases in unemployment in Democratic-, relative to Republican-leaning, counties during the pandemic. Many other potential covariates are netted out because, by modeling within-county fertility change, results are robust to time-invariant factors (e.g., being in south or north Florida) and those that do not differ between Republican- and Democratic-leaning counties (e.g., state COVID-19 regulations).

Racial composition data came from the mid-year Census estimates and unemployment data from the Bureau of Labor Statistics. To parallel the excess fertility measure, both unemployment and racial composition were measured in a similar manner: we created an excess unemployment (or percentage Black or Hispanic) measure that showed, for a given birth quarter, the within-county change in that measure relative to the 2014–2017 average in the same months. The covariates were lagged three quarters to reflect changes observed nine months earlier (i.e., at the time of conception). For example, excess unemployment was the county’s average unemployment rate in 2014–2017 in a specific quarter (e.g., April–June) subtracted from the unemployment rate in the parallel birth quarter (e.g., April–June) and then lagged to the time of conception, demonstrating how at conception the unemployment rate of a county changed over time. Changes in racial composition were based on annual estimates because county-level census estimates were only available yearly (unemployment varies quarterly).

### Analytic Approach

Our basic equation for Model 1 is:

$${\text{Excess}\;\text{Fertility}}_{\text{ct}}=\sum_{t=1}^{18}\beta_{t}\times {\text{Trump}\;\text{Support}}_{c}+\lambda_{t}+\mu_{c}+\epsilon_{\text{ct}}$$

where excess fertility in county *c* and birth quarter time *t* is predicted by 2020 Trump vote share, *Trump Support*_*c*_, in each quarter, *β*_*t*_, time fixed effects, *λ*_*t*_, and county fixed effects, *μ*_*c*_. Time and county fixed effects were included to capture over time trends in excess fertility and time-invariant county differences, respectively.^8^ We used OLS models to estimate coefficients in all 18 quarters except the comparison (omitted) quarter (models with county fixed effects require an omitted category, as models are multicollinear otherwise). We omitted the first quarter in 2018 (t = 1), a pre-pandemic quarter with a small partisan fertility gap (see Table 1). All estimates were normalized to produce a Republican-leaning county–Democratic-leaning county excess fertility gap of zero for the two categorical 2020 Trump support measures or no effect of Trump vote share for the continuous measure in the omitted quarter (a reasonable assumption given the small gap in the first quarter, see Table 1). Thus, in each quarter the coefficient of interest (i.e., *β*_*t*_ × *Trump*_*c*_) shows the change in the partisan gap in excess fertility relative to the first quarter in 2018.

The three measures of 2020 Trump support used here have slightly different interpretations. For the two categorical measures, coefficients show the excess fertility rate difference between majority and supermajority Trump voting counties (i.e., Republican-leaning counties) and counties with less than 50% or less than 60% of Trump support (i.e., Democratic-leaning counties) for a given time *t* relative to the omitted category (January–March 2018). Models that include the categorical measures of 2020 Trump support are difference-in-difference models because they compare the difference between “treated” (i.e., Republican-leaning) and “untreated” (Democratic-leaning) counties before and after the pandemic. For the continuous measure of Trump voting, coefficients reflect how much a 1% point increase in 2020 Trump vote share predicted higher (or lower) excess fertility rates in that quarter relative to the effect in the pre-pandemic omitted quarter (January–March 2018; see Online Supplement Table S2 for estimates without the quarter omitted). These estimates thus indicate the marginal effect of the Trump vote, or how much a 1% point higher Republican vote share predicted a widening or shrinking of the Republican-leaning – Democratic-leaning area fertility gap. Model 2 adds controls for excess Hispanic percentage of the population, excess Black percentage of the population, excess unemployment rate, and interactions between excess unemployment rate and time (we did not find evidence that the effect of racial change measures varied over time). For parsimony, main findings only present results for the Trump support coefficients (estimates for *β*_*t*_), but the full model estimates with excess racial/ethnic composition, unemployment, and time are available in Online Supplement Tables S3–S5. In all models, standard errors are clustered to account for multiple county observations. Models are weighted based on population size. Results from the tables are presented graphically. Figures for the categorical Trump support measures compare counties where Trump received a majority (or supermajority) of 2020 vote share to counties where he received either a minority (or non-supermajority) of votes. For models showing the percent Trump vote share, we predicted excess fertility for low Trump supporting counties (we set this value to 43% of the vote, or the median of counties with less than a majority of Trump vote) and high Trump supporting (set to 68%, or the median of counties with a majority or more of Trump vote). The figures illustrate levels of Republican- and Democratic-leaning area excess fertility over time; below each we plot the partisan gap (for majority or supermajority Trump support, the Republican-leaning – Democratic-leaning counties excess fertility difference and, for percent Trump support, how much a one-point Trump vote share increase predicts changes in excess fertility). If the partisan gap 95% confidence interval (CI) includes zero, it is not statistically significant. If the CI is above (below) zero, then excess fertility in Republican-leaning counties increased (decreased) significantly more than in Democratic-leaning ones. The pandemic period is shaded, with darker shading indicating that conceptions occurred during the pandemic. We also provided all the aforementioned tables and figures stratified by race.

## Results

### Overall Excess Fertility Trends

**Fig. 1:**
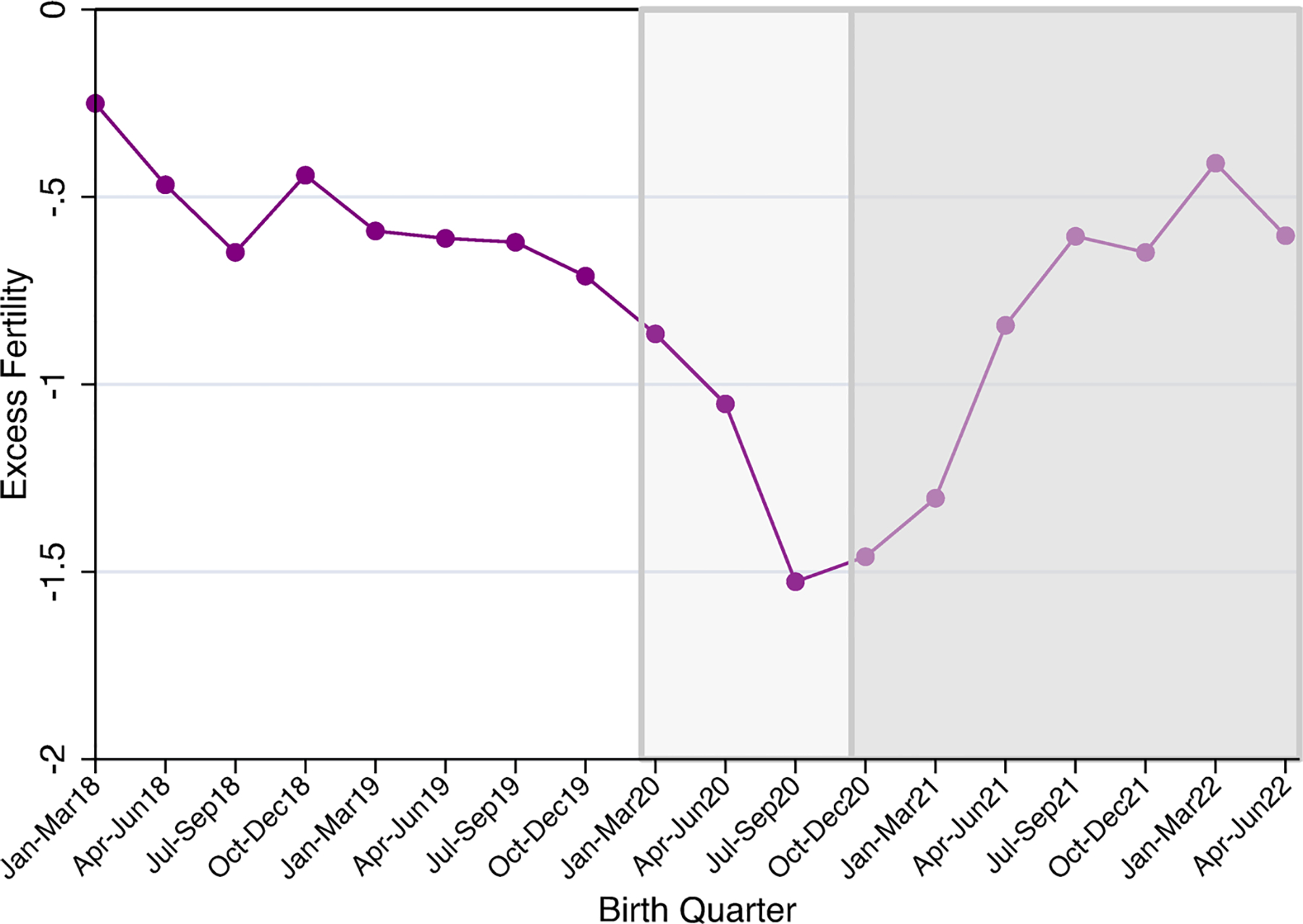
Quarterly excess fertility before and during the COVID-19 pandemic. Excess fertility is calculated as the fertility rate for a quarter (e.g., April–June 2018)—the same season’s (e.g., April–June) 2014–2017 average. The black line (at zero) indicates no change in fertility rate compared to 2014–2017 average fertility. The lighter shaded graph area shows pandemic births and pre-pandemic conceptions. The darker shaded graph area shows pandemic births and conceptions.

We begin by describing excess fertility trends across the years 2018–2022, in which each quarter’s fertility rate was compared to the same quarter’s 2014–2017 average fertility rate (see Table 2; Fig. 1).

Relative to the 2014–2017 period average, the excess fertility measures in 2018–2022 were negative, indicating a decrease in fertility. Declines in excess fertility rates reached their nadir in the third quarter of 2020 (July–September 2020); relative to third quarter rates in 2014–2017, fertility rates were lower by 1.53 births per 1000 reproductive age women. Excess fertility rates remained near this level in October–December 2020 (the first quarter with births conceived during the pandemic) and began rising after that. By the end of our observation period, excess fertility rates appeared to have stabilized and were comparable to their 2018 levels.

**Fig. 2:**
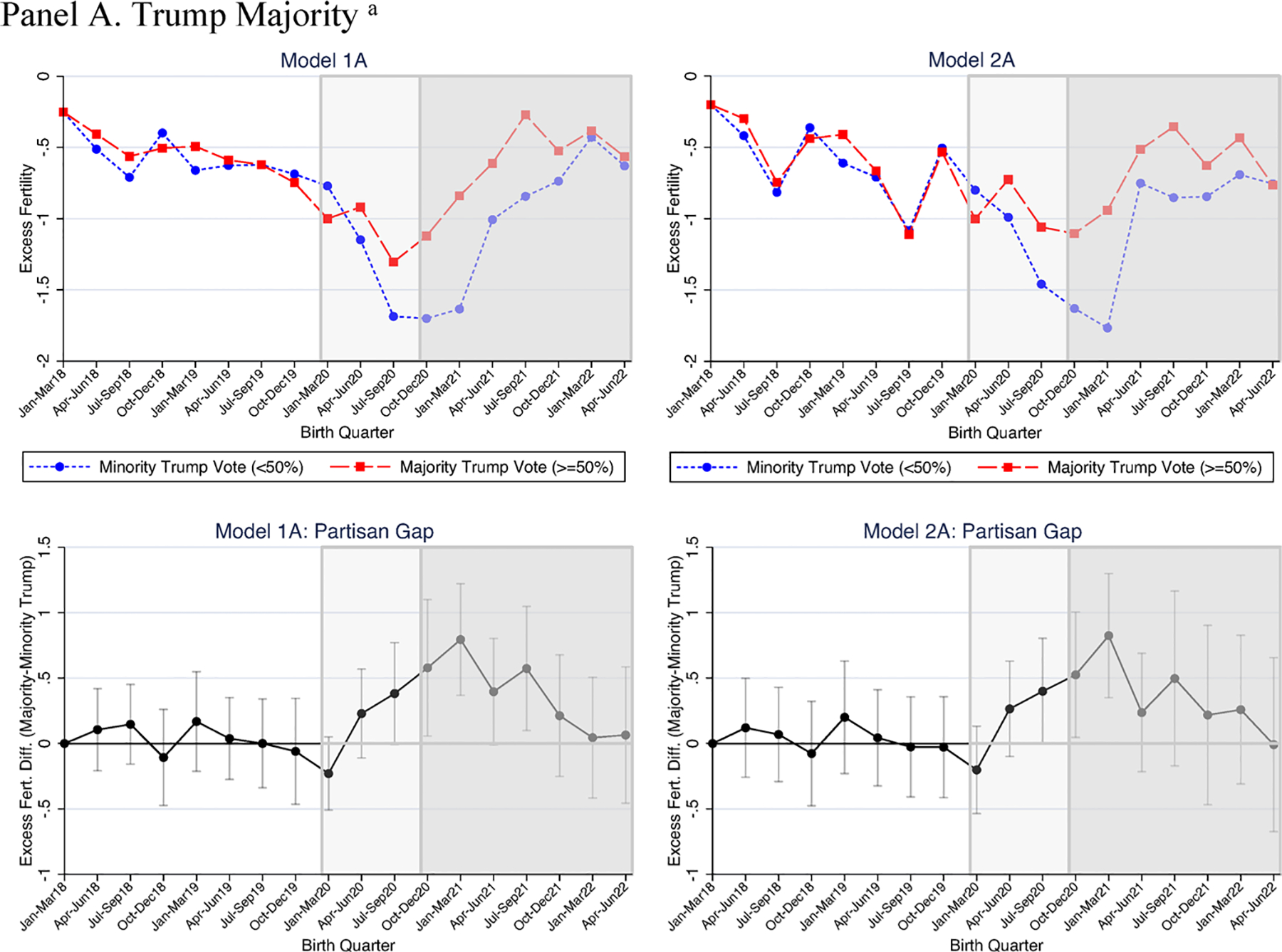

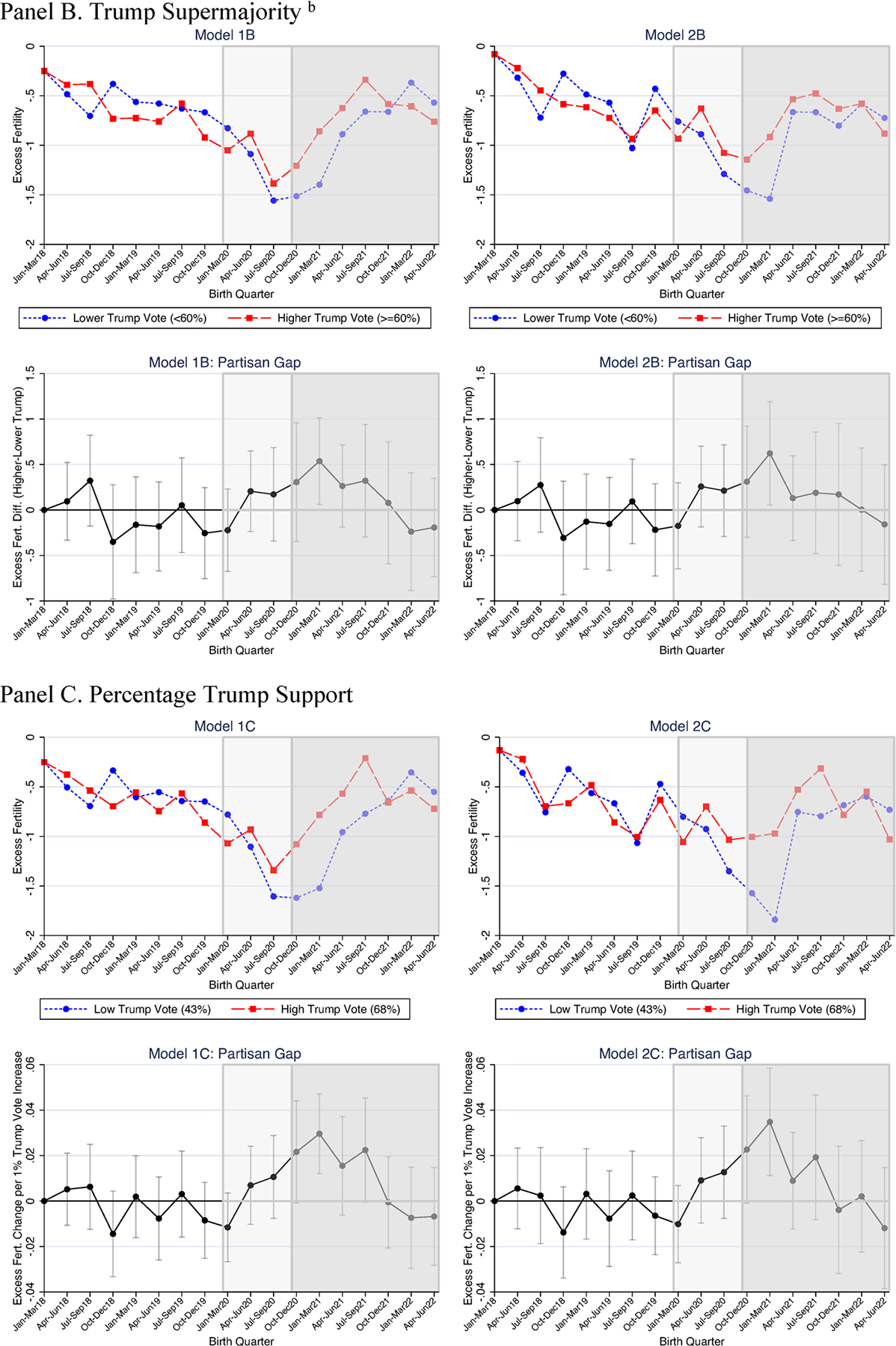
Quarterly excess fertility by 2020 county partisanship. Excess fertility is calculated as the fertility rate in a quarter (e.g., April–June 2018)—the same season’s (e.g., April–June) 2014–2017 average. Estimates from Table 3, where Model 2 controls for county race/ethnicity and unemployment changes and all models include time and county fixed effects. *N* = 56 counties, *N* = 1008 observations. Partisan gap figures show the Republican-leaning county—Democrat-leaning county difference in excess fertility and 95% confidence intervals for that difference. The lighter shaded graph area shows pandemic births and pre-pandemic conceptions. The darker shaded graph area shows pandemic births and conceptions. ^a^Trump majority denotes ≥ 50% Trump 2020 vote share. ^b^Trump Supermajority denotes ≥ 60% 2020 Trump vote share.

Next, to investigate how these declines varied by voting patterns, we consider how the level of Trump support predicted excess fertility rates. As described above, we infer Republican- and Democratic fertility rates based on the partisan county lean using three measures of Trump support in 2020: whether a majority voted for Trump; if Trump received a supermajority of votes (60% or more); and percentage voting for Trump. For each measure of Trump support, the first model includes no controls beyond time and county fixed effects, whereas the second model controls for changes in racial/ethnic composition and unemployment. In Table 3 and Fig. 2 we show estimates of the partisan gap (or change in the excess fertility rate associated with higher, as opposed to lower, levels of 2020 Trump support in a county). The estimates provided in Table 3 are the interaction effects (i.e., partisan gap) from the full models provided in Online Supplement Table S3.

As expected, in the pre-pandemic period (when all conceptions and births were realized pre-pandemic) and in the first three quarters of the pandemic (when conceptions occurred pre-pandemic), 2020 Trump voting level had minimal significant effect on excess fertility in a county (Table 3; Fig. 2).

In the pre-pandemic period (before January 2020), no measure of 2020 Trump support significantly (*p* < .05) predicted fertility changes relative to 2014–2017. Indeed, excess fertility rates were similar in counties that Trump got a majority (or supermajority) of votes in the 2020 election and those where he did not (see Fig. 2). Patterns were similar in the first three quarters of the pandemic, with one exception. In the July–September 2020 quarter, counties where Trump received a majority of votes had excess fertility of 0.4 more than counties where Trump got less than half of votes (*p* < .10, in Model 1 and 2). This marginally significant result was not replicated using our two other measures of Trump support (e.g., percent voting for Trump and Trump supermajority). For the 18 coefficients in the three pandemic birth quarters (18 = 3 quarters × 3 Trump measures × 2 models), two were marginally significant. Overall, we interpret the results to suggest that Trump support was not strongly associated with fertility before pandemic-era births were conceived.

In contrast, with the onset of COVID-19, when some but not all births were conceived after the pandemic began, fertility was higher for Republican-leaning counties than Democratic-leaning counties. In October–December 2020, in models that did not adjust for unemployment or race/ethnicity, two of the three Trump measures predicted significantly higher excess fertility for Republican-leaning counties. Majority Trump support was associated with a 0.58 higher excess fertility rate (*p* < .05; Table 3, Model 1A, and Fig. 2); each percentage point increase in the Trump vote was associated with 0.02 greater excess fertility (*p* < .10; Table 3, Model 1C, and Fig. 2). The other measure, supermajority of votes for Trump (Table 3, Model 1B, and Fig. 2), also indicated higher excess fertility (over 0.3 higher), but this estimate was not statistically significant (*p* = .30).

Differences between Republican- and Democratic-leaning counties’ fertility rates continued to widen during the first quarter of 2021, the first quarter that includes all births conceived during the pandemic. Even after controlling for racial/ethnic and employment changes (Model 2 in Table 3; Fig. 2), all three measures pointed to large and significant partisan differences in the excess fertility rate for births in January–March 2021. A majority and supermajority 2020 Trump vote were associated with 0.82 and 0.62, respectively, higher excess fertility relative to counties without a Trump majority or supermajority (*p* < .05). Similarly, a 1% point increase in 2020 vote share for Trump was associated with 0.035 higher excess fertility (*p* < .05).

To understand the scale of these differences, we can compare the January–March 2021 partisan fertility gap to earlier quarters in the pre-pandemic and pandemic births periods. The estimates for majority Trump vote indicate that the partisan fertility gap was twice as wide in January–March 2021 than in any quarter where births were conceived before the pandemic. Similarly, the marginal effect of Trump vote in January–March 2021 was nearly three times higher compared to the largest coefficient in the pre-pandemic or pandemic births periods. For two of the three measures (majority Trump vote and percentage), estimates were statistically larger than in any prior quarter (except October–December 2020, a quarter that included some pandemic conceptions). The supermajority Trump vote measure was significantly or marginally larger in January–March 2021 than in most previous quarters (exceptions include July–September 2018, July–September 2019, and January–March 2020). The partisan gap for a supermajority of Trump support was at least one and a half times higher in January–March 2021 than in July–September 2018 (the quarter with the widest partisan gap before the pandemic conceptions period). Thus, evidence shows a large and significant partisan excess fertility gap in the first quarter when all births were conceived in the pandemic.

The association between 2020 Trump voting and excess fertility for the last quarter of 2020 and the first quarter of 2021 was statistically robust to the inclusion of racial/ethnic composition and unemployment (i.e., the partisan gap did not attenuate, see Table 3). However, the inclusion of unemployment, specifically, altered the predicted over-time trend lines. Relative to the last quarter before the pandemic conceptions period (July–September 2020), excess fertility in counties with high 2020 Trump support began an upward trend in the first quarter that included pandemic conceptions (October–December 2020) in unadjusted models (e.g., Models 1) (Fig. 2).^9^ After accounting for changes in unemployment (Model 2), however, the Republicanleaning area increases in the first two pandemic conceptions quarters were less pronounced (see Fig. 2, Panels A and B, Models 2A and 2B) or seemingly plateaued (see Fig. 2, Panel C, Model 2C). Democratic-leaning area’s excess fertility plateaued in the first two pandemic conceptions quarters, but exhibited downward trends once unemployment was accounted for (see Fig. 2). This change was observed because, for counties of both political leans, excess fertility would have been expected to be more negative (less negative) in January–March 2021 (in July–September 2020) than it was given the unemployment rise (fall) and negative association between unemployment and fertility. Thus, including unemployment results in less of an upward trend for Republican-leaning and more of a downward one for Democratic-leaning counties between July–September 2020 and January–March 2021. Nevertheless, the predicted relative size of the partisan gap, for the last quarter of 2020 and the first quarter of 2021, was actually larger in models that adjusted for unemployment relative to those that did not.

The strong 2020 Trump vote share-fertility relationship was short lived. Beginning in the second quarter of 2021 (April–June), Trump support did not show robust associations with excess fertility rates because Democratic-leaning area fertility increased sharply, while Republican-leaning area fertility continued a steady climb. For instance, in Fig. 2, Panel A, Model 1A, Democratic-leaning area excess fertility rose by 0.6 in January–March 2021 to April–June 2021 (−1.6 to −1), whereas Republican-leaning area fertility rose by 0.2. After January–March 2021, Trump support was only associated with higher fertility in a few quarters and all these associations were explained by the inclusion of covariates (see Table 3). In April–June 2021, a Trump majority predicted marginally higher excess fertility (reflecting a relatively wide partisan gap even after Democratic-leaning fertility rose steeply because Republican-leaning fertility steadily increased since July–September 2020, see Table 3; Fig. 2, Panel A, Model 1A), an association that became nonsignificant once models included racial and unemployment changes. Similarly, in July–September 2021, the first quarter that included some births conceived after Biden’s election (all conceived during the pandemic), the significant increase in excess fertility associated with Trump voting measures was accounted for by changes in unemployment and race (see Table 3). Beyond September 2021, Republican-leaning and Democratic-leaning counties exhibited fertility that was substantively and statistically similar. Interestingly, Biden’s election (reflected in the final three to four quarters), corresponded with a reversal of the steady climb in excess fertility for Republican-leaning counties that began before and continued during the earlier pandemic quarters.

### Excess Fertility Trends by Race

We now turn to estimates of the partisan gap by race. As shown above, we provide only the partisan gap over time in Tables 4 (for White women) and 5 (for non-White women). For the full models from which these estimates were derived see Online Supplement Tables S4 and S5, respectively.

In the pre-pandemic period, results varied little by race. For both White and non-White women’s fertility, associations between the Trump support measures and excess fertility were small and generally nonsignificant (see Tables 4 and 5).

Within each group, only two coefficients were marginally significant out of the 42 examined (7 pre-pandemic quarters × 3 Trump support measures ×2 models), with only two instances where the fertility estimate for White women differed at conventional levels of statistical significance (*p* < .10) from that of non-White women (supermajority of Trump support in the first quarter of 2019 in Tables 4 and 5, Models 1B and 2B). Overall, evidence indicates that neither White or non-White women’s excess fertility were associated with Trump support in the pre-pandemic period.

In the pandemic births period (January–March 2020 to July–September 2020), estimates did not differ significantly by race. Among White women, results did not indicate a consistent set of statistically significant findings for the three 2020 Trump support measures across time. In January–March 2020, for example, the continuous measure of Trump support predicted declines in Republican-leaning area fertility, but the other two measures were small and non-significant (see Table 4). In the next two quarters (April–June 2020 and July–September 2020) majority Republican-leaning, versus Democratic-leaning, counties had higher excess fertility rates for White births, but again we found no significant association for the other two measures (here Trump supermajority and percentage vote).^10^ For non-White women, however, the Trump support measures were not significantly predictive of excess fertility during the time when babies were born but not conceived in the pandemic. Additionally, models indicated no significant racial differences in the associations between county partisanship and fertility in any model or operationalization of Trump support.

**Fig. 3:**
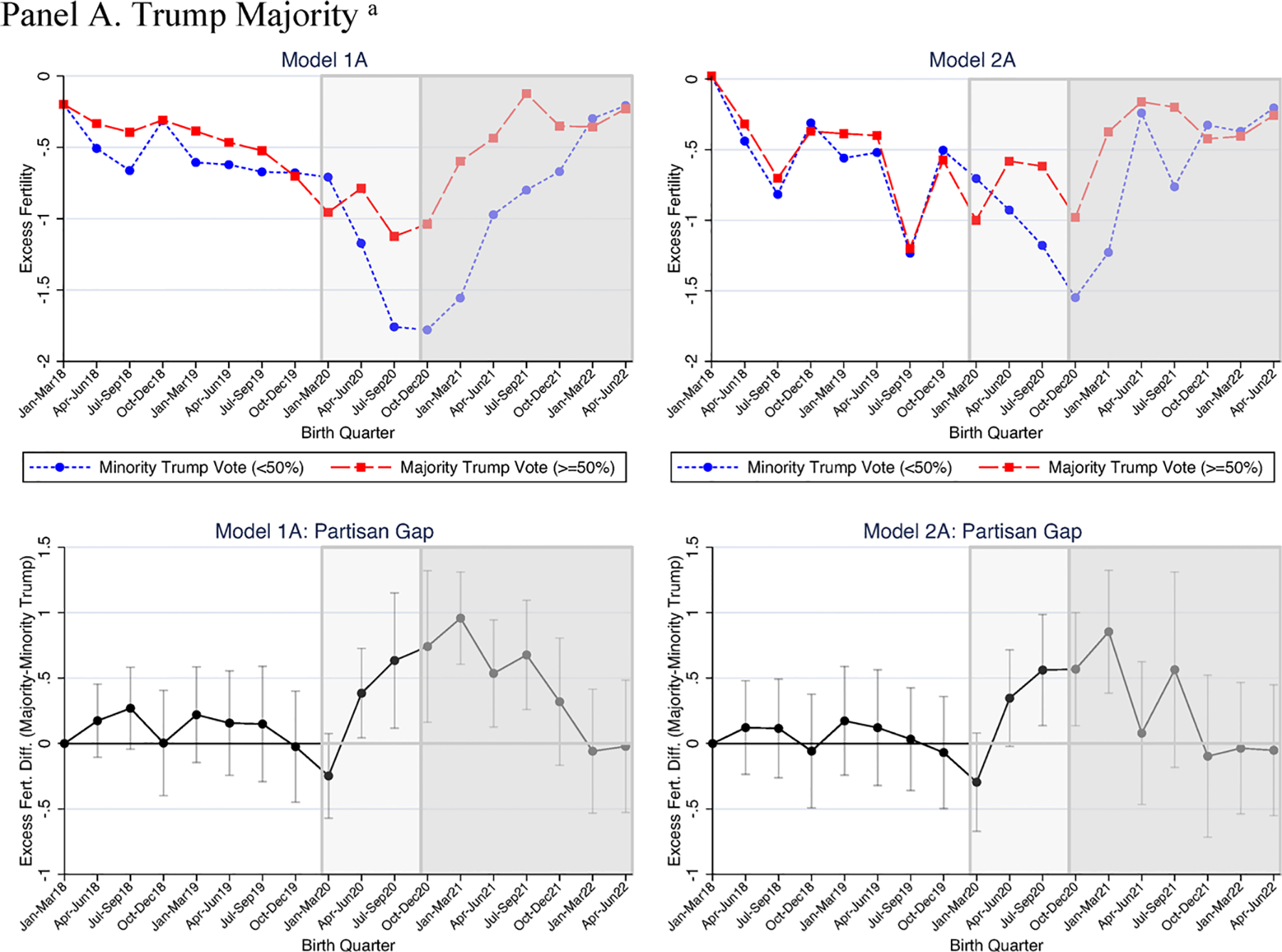

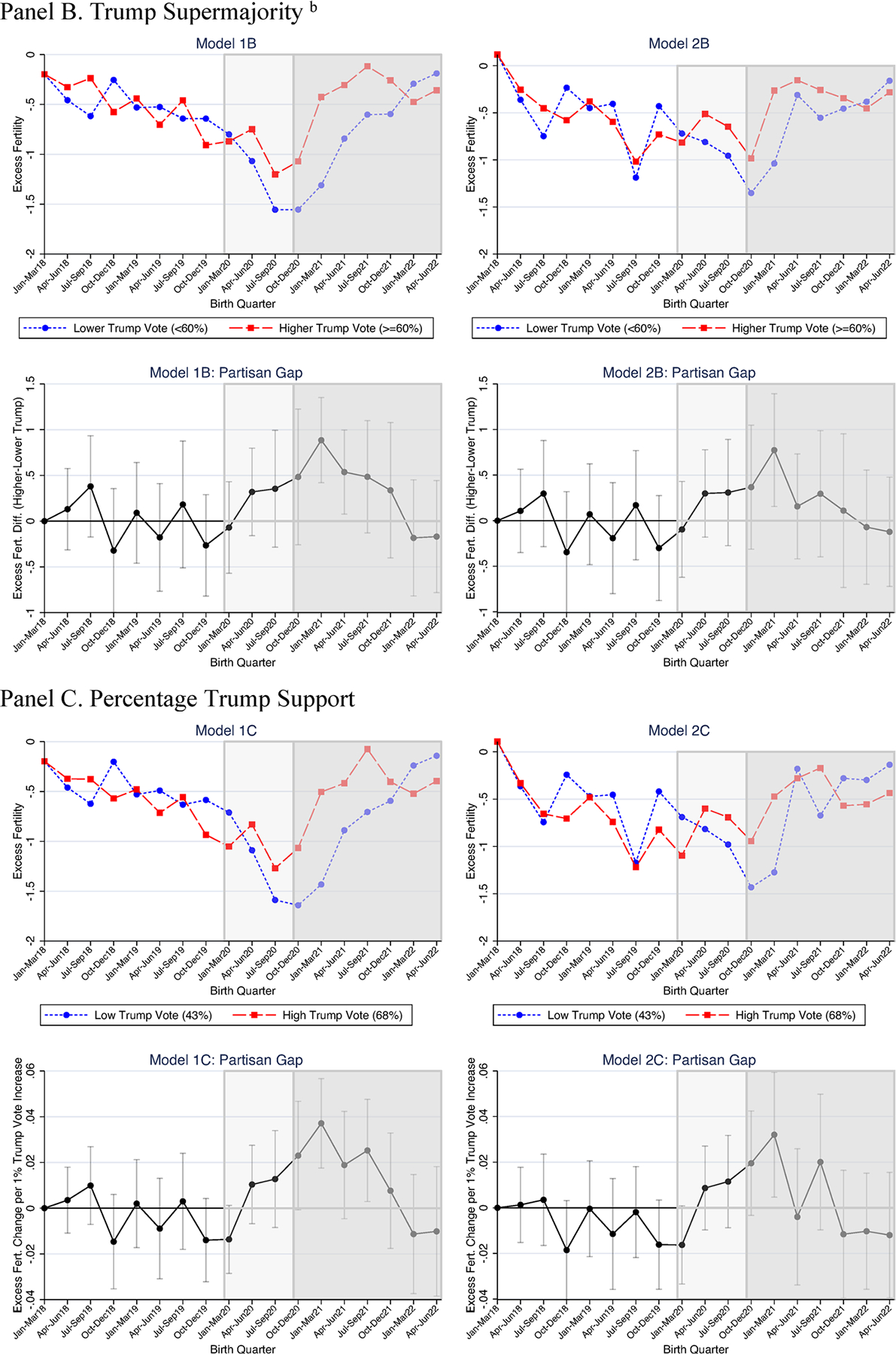
Quarterly excess fertility by 2020 county partisanship, White women. Excess fertility is calculated as the fertility rate in a quarter (e.g., April–June 2018)–the same season’s (e.g., April–June) 2014–2017 average for White women. Estimates from Table 4, where Model 2 controls for county race/ethnicity and unemployment changes and all models include time and county fixed effects. *N* = 55 counties, *N* = 990 observations. Partisan gap figures show the Republican-leaning county–Democrat-leaning county difference in excess fertility and 95% confidence intervals for that difference. The lighter shaded graph area shows pandemic births and pre-pandemic conceptions. The darker shaded graph area shows pandemic births and conceptions. ^a^Trump majority denotes ≥ 50% Trump 2020 vote share. ^b^Trump Supermajority denotes ≥ 60% 2020 Trump vote share.

In the early pandemic conceptions period (e.g., January–March 2021), however, excess fertility among White women, relative to non-White women, may have been more responsive to Trump support. Among White women, in unadjusted models, all three Trump support measures predicted significantly higher excess fertility in the first full pandemic conceptions quarter (Table 4; Fig. 3).

**Fig. 4:**
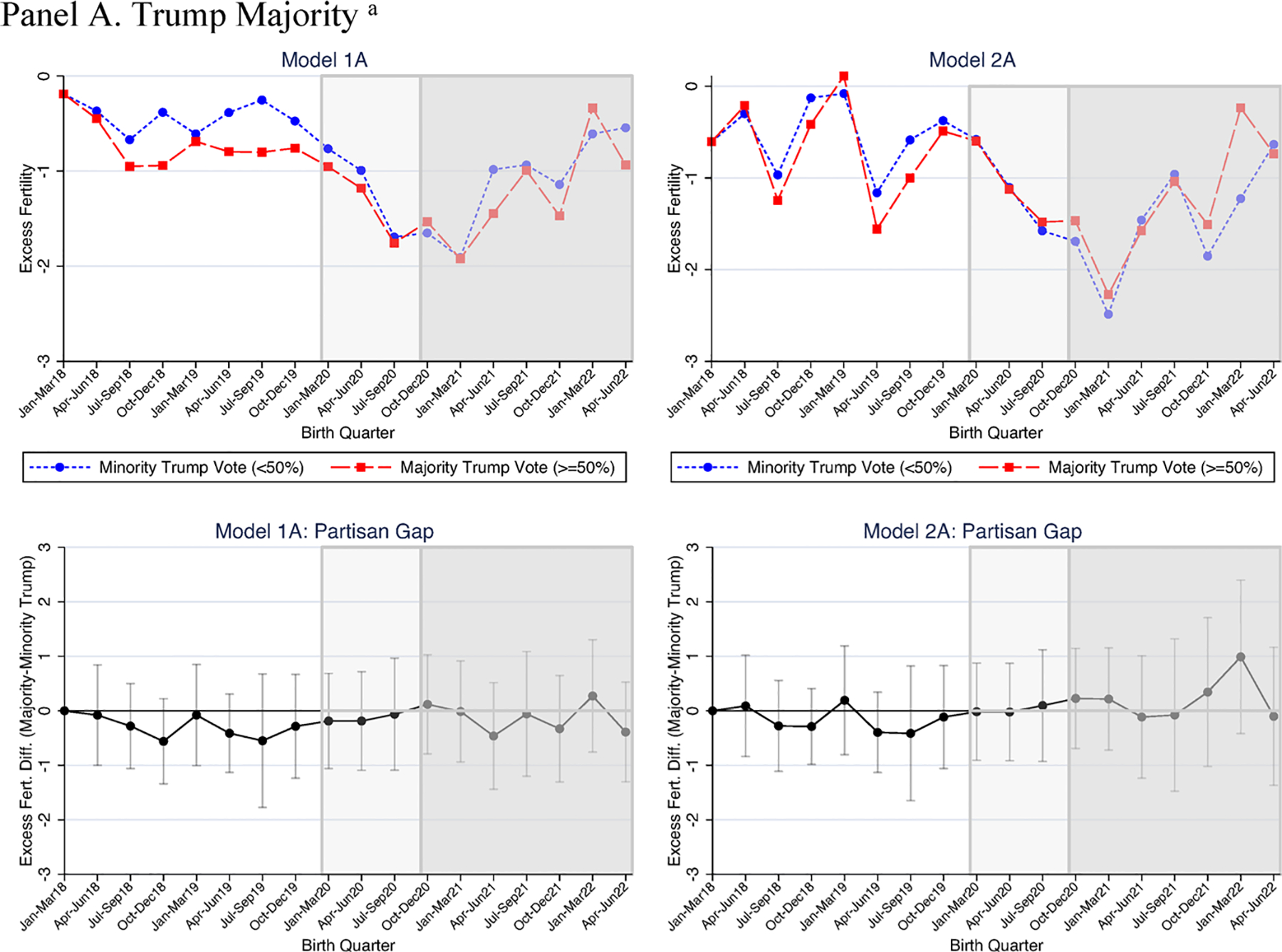

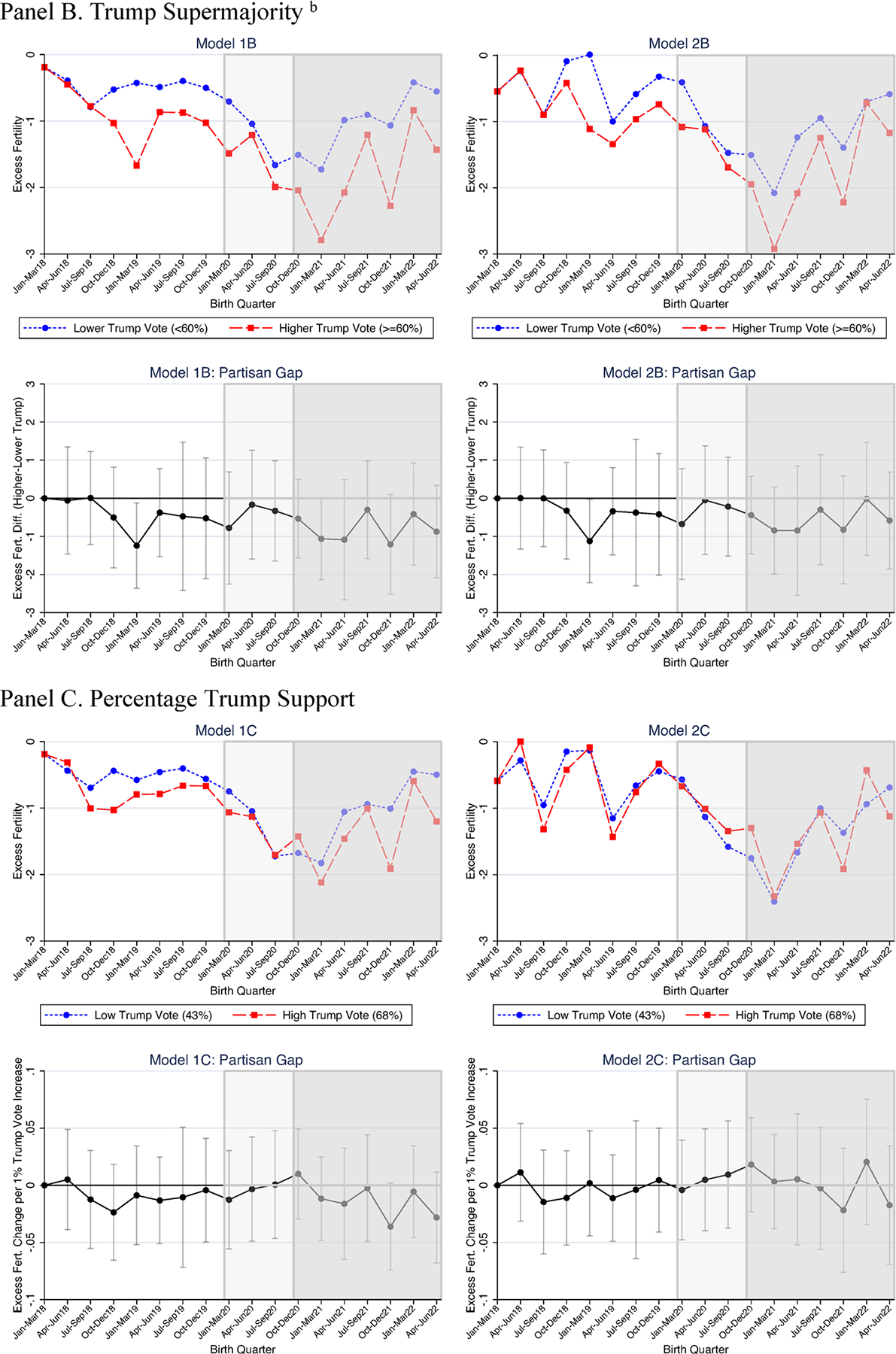
Quarterly excess fertility by 2020 county partisanship, non-White women. Excess fertility is calculated as the fertility rate in a quarter (e.g., April–June 2018)–the same season’s (e.g., April–June) 2014–2017 average for non-White women. Estimates from Table 5, where Model 2 controls for county race/ethnicity and unemployment changes and all models include time and county fixed effects. *N* = 55 counties, *N* = 990 observations. Partisan gap figures show the Republican-leaning county–Democrat-leaning county difference in excess fertility and 95% confidence intervals for that difference. The lighter shading graph area shows pandemic births and pre-pandemic conceptions. The darker shading graph area shows pandemic births and conceptions. ^a^Trump majority denotes ≥ 50% Trump 2020 vote share. ^b^Trump Supermajority denotes ≥ 60% 2020 Trump vote share.

Estimates indicated quite large partisan gaps: for example, the 0.96 estimate associated with majority Trump support (Table 4; Model 1A) is seven times higher than the average gap in the pre-pandemic period (i.e., 0.135) and nearly four times higher than the average gap in the pandemic births period (0.257). All three measures were significant predictors of the excess fertility gap after adjusting for unemployment and racial composition (see Table 4, Model 2). In contrast, among non-White women (Table 5; Fig. 4), both the majority and continuous Trump measures indicated small and nonsignificant Republican-Democratic fertility gaps (e.g., −0.01 for January–March 2021; Table 5, Model 1C), as those in both Republican- and Democratic-leaning counties exhibited similarly sized fertility rate declines relative to pre-pandemic fertility. The other Trump support measure, a supermajority of votes, was in the opposite direction for non-White women’s fertility (−1.06, *p* < .10, Table 5, Model 1B) relative to White women’s fertility (vs. 0.89, *p* < .001; Table 4, Model 1B). The non-White coefficient reduced in size and was no longer statistically significant after adjusting for racial composition and unemployment. Statistical tests indicated that in the unadjusted estimates (Model 1), for White women the coefficients associated with all three Trump support measures were significantly larger (*p* < .05) than those for non-White women. In adjusted estimates (Model 2), two of the three Trump support measures differed by race at conventional levels of statistical significance (percent Trump support did not differ by race, *p* = .127). Nevertheless, racial differences in the marginal effect of Trump vote share were substantively large: for example, in January–March of 2021, the effect of each 1% increase in Trump vote on excess fertility rate for White women was 0.032 (Table 4, Model 2C), ten times higher than for nonWhite women, 0.003 (Table 5, Model 2C).

In two later pandemic conception quarters (April–June 2021 and October–December 2021) evidence for racial variation in unadjusted models was explained by controlling for changes in unemployment. For unadjusted models, in April–June 2021, Trump support measures predicted significantly higher excess fertility for White women (for two out of the three measures), but none of the measures predicted higher non-White women’s fertility; and in unadjusted models all three Trump support measures were significantly higher when predicting White vs. non-White women’s fertility (see Tables 4 and 5). After controlling for unemployment, all coefficients were substantially smaller and there was no evidence for significant racial differences. Similarly, in October–December 2021 findings suggested racial variation in unadjusted models for two Trump support measures, but this was statistically accounted for by the inclusion of unemployment (see Model 2).

Interestingly, for White women, the Trump effect changed directions for births conceived after Biden took office and the insurrection (see Fig. 3). In both unadjusted and adjusted models, after about a year of steady increases and higher excess fertility, the Republican-leaning area excess fertility rate climb halted or reversed direction after Biden took office (i.e., January 2021 conceptions reflect October 2022 births). In contrast, Democratic-leaning area fertility increases continued and became slightly higher than Republican-leaning area fertility for the first time in over a year and a half.

In sum, Trump support differentially affected White versus non-White women’s excess fertility in the early months of the pandemic. Prior to the pandemic, Trump support mattered little for either White or non-White women’s excess fertility. In the first full quarter with pandemic conceptions, however, among White women, excess fertility was much higher in Republican- rather than Democratic-leaning counties; non-White women’s fertility exhibited no such differences. As with the full sample results, excess fertility associated with the Trump vote faded relatively quickly for White women. Nevertheless, results suggest an early pandemic period during which Trump support predicted an increase in births to White women over what would have been expected given pre-pandemic averages.

### Sensitivity Analysis

We did several robustness tests. We used the 2016 Trump and 2018 DeSantis (Republican) governor’s race vote shares to predict excess fertility; results were nearly identical to those shown here (as the 2020 Trump vote was highly correlated with the 2016 vote [ρ = .95] and the 2018 DeSantis vote [ρ = .99]). We also tested whether COVID-19 severity confounded our results. Using data on county cumulative (or new) COVID-19 case rates in each conception quarter (NYT, 2021), we found that partisan fertility gaps were not explained by controlling on case rates.

We also explored if the larger partisan gaps for White women’s fertility than non-White women’s fertility reflected marital, rather than racial, differences insofar as White women are more likely to be married at birth (Schweizer, 2020). We compared the January–March 2021 partisan gaps by marital status within each racial group. For White women, the partisan excess fertility gaps were larger when predicting marital rather than non-marital fertility rates.^11^ One explanation is that the political context may have a greater effect on the intention to have a child and marital births are more likely to be intended (Mark & Cowan, 2022). Among non-White women, however, there was no difference in the January–March 2021 partisan gap by marital status.^12^ For non-White women, Republican- to Democratic-leaning area fertility gaps were either small or negative regardless of marital status. Perhaps Trump support did not predict marital or non-marital fertility for non-White women because the political context did not reflect their own or their close associates’ politically informed views. Thus, at least for White women, evidence suggests the early pandemic partisan fertility gap was larger when predicting marital (and perhaps intended), rather than non-marital, births.

We then examined if education explained the partisan gap, to explore if socioeconomic advantage was a more salient context than 2020 Trump support. After controlling for percent without a high school degree, with a high school degree, a B.A. or more and their interactions with time,^13^ we found that the partisan gap was robust for a majority and percent of Trump support in the first quarter of all pandemic conceptions (January–March 2021), albeit education explained the partisan gap for a Trump supermajority (see Online Supplement Figure S1). But we note that education and Trump voting at the county level are highly correlated (*r* = .70), so it is difficult to identify if we are picking up a unique effect of education.

In another robustness check, we conducted Seasonal Autoregressive Integrated Moving Average (SARIMA) modeling. SARIMA allows us to analyze deviations from forecasted trends, making it an excellent tool to understand if pandemic fertility was a departure from pre-pandemic trends (unfortunately, SARIMA models do not permit interaction terms, meaning that we cannot use it to analyze differences by 2020 Trump support).^14^ We used trends in quarterly birth counts between 2014 and 2019 to establish trends and then examined deviation from those counts for 2020–2022 for all counties (see Online Supplement Figure S2).^15^ We repeated this procedure separately for counties with and without a Trump majority (see Online Supplement Figure S3). Results showed that fertility changes during the pandemic for non-Trump majority counties (i.e., Democratic-leaning) were significantly more negative than forecasted. These declines in fertility, moreover, were larger than the declines in counties where Trump received a majority of the 2020 vote share. In January–March 2021, minority Trump vote counties deviated significantly from forecasts (-8%) but Trump majority counties only deviated slightly and nonsignificantly (0.9%). Fertility deviations in Democratic-leaving counties returned to baseline by April 2021. Only Trump majority counties saw a fertility rebound from July 2021 onward. Thus, a modest but real fertility decline occurred early in the pandemic for Democratic-leaning counties and a later rebound occurred in Republican-leaning ones.

We further investigated if the early pandemic excess fertility decline in Democratic-leaning counties reflected pre-pandemic trends by comparing percent change in fertility rates relative to the prior year’s same quarter (e.g., [January–March 2021 – January–March 2020]/January–March 2020). In October–December 2020 and January–March 2021, relative to the same quarter in the prior year, fertility was about 8% lower for non-Trump majority counties. Compare this 8% decline to the 2.7% drop (in October–December 2020) and 1.3% nonsignificant increase in (in January–March 2021) in Trump majority counties (Online Supplement Figure S4). The partisan gap in both quarters was statistically significant (i.e., relative to the prior year, fertility dropped more in Democratic-leaning counties). In all quarters reflecting pre-pandemic conceptions, however, the average percent year-to-year change was only a 2.6% drop for non-Trump majority counties and 1.9% drop for Trump majority ones (and the partisan gap was rarely significant). Like above, early in the pandemic Democratic-leaning counties had a larger decline in their fertility rate relative to before the pandemic. This decline was also larger than that in Republican-leaning counties.

## Discussion and Conclusion

Motivated by evidence suggesting that the hyper-partisanship exhibited in the early months of the pandemic led to partisan gaps in both attitudes and behaviors (Allcott et al., 2020; Fan et al., 2023; Gadarian et al., 2021), our study analyzed whether the onset of the COVID-19 pandemic led to an increased partisan gap in fertility. Consistent with expectations, we found large significant partisan fertility gaps for births conceived early in the pandemic for all three measures of 2020 Trump vote share. For instance, in January–March 2021, for the majority Trump support measure, partisan fertility gaps were over 19 times higher than the average pre-pandemic partisan gap (i.e., a 0.80 gap in January–March 2021 vs. a 0.04 average gap in the pre-pandemic period). These divergent patterns arose because, relative to pre-pandemic levels, Republican-leaning counties exhibited less fertility decline than did Democratic-leaning counties. After adjusting for changes in unemployment, increases in Republican-leaning counties’ fertility were more muted and declines in Democratic-leaning counties’ fertility were more dramatic. Thus, our results suggest that, with the onset of the pandemic, the partisan fertility gap widened sharply.

Nonetheless, consistent with other work (Nobles et al., 2024), changes in Democratic-leaning counties were modest and short-lived; even though these drops continued a trend of fertility decline, the pandemic drops were significantly higher for Democratic-leaning counties than Republican-leaning ones. Excess fertility declined by 1.7 for minority-Trump and 0.9 for majority-Trump counties in the first quarter with all pandemic conceptions (January–March 2021). SARIMA models that take prior trends into account showed larger fertility deviations than forecasted early in the pandemic in Democratic-leaning counties than in Republican-leaning ones; later in the pandemic, deviations in Democratic-leaning counties quickly returned to the forecasted trends while Republican-leaning counties exceeded forecasts.

Results showed early COVID-19 pandemic partisan gaps were robust to different modeling strategies and measures of 2020 Trump support. We found an early pandemic partisan gap in fertility changes using difference-in-difference models of excess fertility rates, SARIMA models, and year-over-year percent changes suggesting there was a differing fertility response by county-level 2020 Trump support. Further, all three 2020 Trump support variables gave similar results, showing incremental increases in 2020 Trump vote share (vs. a nonlinear effect, e.g., a tipping point or threshold effect), mattered and provides confidence that results were not idiosyncratic to measurement of Trump support. This cohesive set of results provides greater evidence of a true, albeit modest, linear association between Trump support and early pandemic changes in fertility.

Although we cannot test the mechanisms that led to these diverging responses, we speculate on potential pathways through which the COVID-19 pandemic influenced a partisan gap in fertility. Fertility decision-making may be shaped by the interaction of material contexts and cognitive meanings of the world and imagined futures that are informed by social contexts (Johnson-Hanks et al., 2011; Marshall & Shepherd, 2018; Vignoli et al., 2020). For instance, among Republicans or Democrats, or those predominantly surrounded by partisans, material conditions of job, income, and childcare instability—despite fiscal supplements—may have been interpreted through distinct ideological lenses shaped by cognitive notions of ideal family structures and optimism for a return to pre-pandemic normalcy. Republicans, who tend to support traditional gender roles, might have perceived disruptions to women’s employment as reinforcing an ideal family model, where women could adopt full-time caregiving roles. The combination of this outlook with enhanced fiscal support and a belief in Republican leadership’s capacity to manage the crisis effectively could have bolstered a positive view of the future, where instability seemed temporary and manageable. In such contexts, childbearing decisions would likely be seen as viable, even in uncertain times, as the alignment between traditional family roles and optimism for recovery alleviated concerns over long-term instability.

Conversely, Democrats, who generally support egalitarian gender roles, may have viewed the same uncertainties as fundamentally at odds with a model of shared economic and caregiving responsibilities, rendering the pandemic’s instability less compatible with their family ideals. The pandemic, from this perspective, underscored not only immediate challenges but also signaled potential long-term volatility. Democrats’ skepticism toward the Republican-led government’s crisis management likely compounded this perception, fostering negative and uncertain views of the future and postponement of childbearing. Thus, the optimism observed among Republicans—rooted in traditional family expectations and trust in recovery—may have softened early pandemic fertility declines, while Democrats’ concerns over sustained instability and discordance with egalitarian ideals likely contributed to postponement of fertility. These speculations are consistent with studies showing partisanship shapes interpretation of material contexts and likely fertility (Comolli & Andersson, 2021; Dahl et al., 2022; Morgan et al., 2011).

These county-level partisan gaps in fertility were short-lived. By July 2021, partisan gaps had dissipated as excess fertility increased in both Republican- and Democratic-leaning counties. Our findings showed that the partisan gap narrowed because of the continued gradual Republican-leaning counties’ increase in excess fertility and steep increase in Democratic-leaning counties’ excess fertility relative to the first quarter where all births were conceived during the pandemic (January–March 2021). We can only speculate as to why the early pandemic partisan gap attenuated, but perhaps the material context of improving employment (Falk et al., 2021) and household wealth (Aladangady et al., 2023) combined with the fading anxiety surrounding the uncertainty of the pandemic (as the initial shock of the pandemic abated and with vaccines on the horizon and Biden’s election) led to fertility in Democratic-leaning counties increasing and catching up with fertility in Republican-leaning ones.

Partisan fertility gaps generally were not evident before 2020. People may look to thought leaders, media, and those around them during times of uncertainty to understand what unexpected events mean for them and their future (Vignoli et al., 2020). When there is less uncertainty, however, these influences are less relevant because people feel more confident anticipating what their future will look like based on their own prior experiences or those of their peers. Unexpected circumstances that change views about the future might be more relevant for activating fertility responses. For instance, in two presidential elections with unexpected and/or uncertain outcomes (i.e., 2000 Bush vs. Gore and 2016 Trump vs. Clinton), there was county-level divergence in fertility changes with supporters of the winner showing less declines; but no divergence with expected wins (i.e., Obama 2008; see Dahl et al., 2022). Perhaps fertility responses depend on the narratives of the current and future situation shared by important social others especially in unexpected circumstances.

We also anticipated that Trump support would have different effects for White, as opposed to non-White, women, as we believed county-level Trump support would be a better proxy for the politically informed milieu of White than non-White adults. Results were consistent with this, as we found no positive Republican-Democratic leaning county fertility gaps for non-White women but did for White women. Like the primary results, White women exhibited a partisan gap early in the pandemic that soon dissipated. Effects for White women’s excess fertility were large: during the first quarter where all babies were conceived during the pandemic (January–March 2021), the partisan gap was larger than nearly all quarters where babies were conceived prior to the pandemic.

Although the stronger effect of Trump support on White women’s, opposed to non-White women’s, fertility suggests that partisan views about the pandemic are important for fertility decisions, other factors may contribute to these racial differences. For example, racial groups may respond differently to the same policies due to variations in residential arrangements, which can shape the experience and impact of confinement. Also, school closures may affect families unevenly, depending on differences in work schedules and financial resources. Future research that incorporates both individual partisan identity and fertility could help disentangle these complex relationships.

This study has limitations. First, Florida is one state and results may not generalize to other states. But using Florida as a study context has several positive aspects (e.g., large populations and quickly available data). Additionally, trends shown here (fertility decline and quick rebound) are consistent with larger U.S. trends (Bailey et al., 2023). Future research should extend this analysis to national data sources, particularly the National Vital Statistics System (NVSS), to assess whether these patterns and associations hold across wider geographic areas and racial/ethnic groups. NVSS data would allow for easier identification of birth order and other characteristics that may be associated with different fertility responses during Republican and Democratic presidencies. But early in the COVID-19 pandemic micro-level NVSS data was not available and publicly accessible NVSS data does not include county of birth (other data sources like CDC’s WONDER only provides birth counts in counties with more than 100,000 residents) making identifying Trump vote share in counties of birth difficult. Second, we only included data through June of 2022. Future work should examine longer time frames than was possible here, which would be important to further disentangle the effects of partisan alignment, presidential administration, and the unfolding of the COVID-19 pandemic on fertility behavior. For example, an analysis could compare fertility rates by partisan vote share between the first Trump presidency during the COVID-19 pandemic and the mid-2020s during the second Trump presidency. This approach would hold constant the effect of having a Republican in the White House while identifying the effect on fertility rates that were attributed to the pandemic.

Third, we only observed aggregate-level voting behavior and fertility change. Nevertheless, our results are consistent with individual-level work showing political identity predicts behavior, beliefs, and changes in fertility intentions during the pandemic (Allcott et al., 2020; Klofstad et al., 2013; Margolis, 2022; Mason, 2018; Wilcox et al., 2021). Further, like others (Dahl et al., 2022; Gollwitzer et al., 2020) we use aggregate-level data because, as far as we know, no other data has prospective measures of individual political identity and fertility measured multiple times per year. Indeed, surveys used by fertility researchers (e.g., NSFG) generally do not collect information on political identity, but we hope that more surveys will consider adding partisan affiliation because it may be important for fertility (Rackin & Gibson-Davis, 2024). Fourth, we cannot explore individual-level fertility decision making processes. Our results do align, though, with other individual-level and aggregate-level findings linking views and fertility during uncertain times (Comolli & Vignoli, 2021; Guetto et al., 2020, 2023; Lappegård et al., 2022; Vignoli et al., 2022). Fifth, we cannot explicitly model either proximate mechanisms (e.g., sexual intercourse, contraception) or the more distal determinants (e.g., one’s own, or close associates’, COVID-19 pandemic views) underlying this relationship, though we suggest this is a fruitful area for future research.

Despite these limitations, our study provides evidence that the political context may influence fertility responses perhaps especially when an unexpected event occurs and brings uncertainty. We add to a growing literature that demonstrates the salience of partisanship for behavior (Allcott et al., 2020; Coffey & Joseph, 2013; Gadarian et al., 2021) but has rarely considered fertility. By revealing that partisan context impacts even deeply personal and consequential decisions, like whether to have a child, this research underscores the need to integrate political context into demographic research. This study helps us grasp why changes in fertility occur, an urgent endeavor given that U.S. fertility has reached an all-time low and political polarization has reached a record high (Hamilton et al., 2021; Layman et al., 2006).

## Supplementary Material

Supplement Materials

**Supplementary Information** The online version contains supplementary material available at https://doi.org/10.1007/s11113-025-09972-0.

## Figures and Tables

**Table 1:** Time period, birth quarter, and conception quarter

Time period	Birth Quarter	Conception Quarter
Pre-pandemic birth and conception	Jan–Mar 2018	Apr–Jun 2017
	Apr–Jun 2018	July–Sep 2017
	July–Sep 2018	Oct–Dec 2017
	Oct–Dec 2018	Jan–Mar 2018
	Jan–Mar 2019	Apr–Jun 2018
	Apr–Jun 2019	July–Sep 2018
	July–Sep 2019	Oct–Dec 2018
	Oct–Dec 2019	Jan–Mar 2019
Pandemic birth, but not conception	Jan–Mar 2020^a^	Apr–Jun 2019
	Apr–Jun 2020	July–Sep 2019
	July–Sep 2020	Oct–Dec 2019
Pandemic birth and conception	Oct–Dec 2020^b^	Jan–Mar 2020
	Jan–Mar 2021	Apr–Jun 2020
	Apr–Jun 2021	July–Sep 2020
	July–Sep 2021	Oct–Dec 2020
	Oct–Dec 2021	Jan–Mar 2021
	Jan–Mar 2022	Apr–Jun 2021
	Apr–Jun 2022	July–Sep 2021

Months are abbreviated;

a Quarter contains some pre-pandemic births and some pandemic births;

b Quarter contains some pandemic births (but not conceptions) and some pandemic conceptions

**Table 2:** Quarterly fertility rate and partisan gap, by conception and birth time period before and during the COVID-19 pandemic

Time period	Birth quarter	Fertility rate	Excess fertility rate^a^	Partisan	
				Fertility rate gap^b^	Excess fertility rate gap^c^
Pre-pandemic birth and conception	Jan–Mar 2018	11.99 (1.14)	− 0.25 (0.40)	0.35	− 0.06
	Apr–Jun 2018	11.57 (1.25)	− 0.47 (0.52)	0.52	0.05
	Jul–Sep 2018	12.71 (1.36)	− 0.65 (0.55)	0.73*	0.09
	Oct–Dec 2018	12.64 (1.25)	− 0.44 (0.64)	0.3	− 0.16
	Jan–Mar 2019	11.65 (1.24)	− 0.59 (0.60)	0.52	0.11
	Apr–Jun 2019	11.43 (1.06)	− 0.61 (0.50)	0.45	− 0.02
	Jul–Sep 2019	12.74 (1.44)	− 0.62 (0.62)	0.58 ^†^	− 0.06
	Oct–Dec 2019	12.37 (1.34)	− 0.71 (0.60)	0.34	− 0.12
Pandemic birth, but not conception	Jan–Mar 2020	11.38 (1.09)	− 0.87 (0.54)	0.12	− 0.29 ^†^
	Apr–Jun 2020	10.99 (1.19)	− 1.05 (0.56)	0.63 ^†^	0.17
	Jul–Sep 2020	11.83 (1.28)	− 1.53 (0.57)	0.95**	0.33*
Pandemic birth and conception	Oct–Dec 2020	11.62 (1.42)	− 1.46 (0.72)	0.97**	0.52**
	Jan–Mar 2021	10.94 (1.29)	− 1.30 (0.64)	1.13***	0.74***
	Apr–Jun 2021	11.20 (1.26)	− 0.84 (0.52)	0.79*	0.34*
	Jul–Sep 2021	12.75 (1.43)	− 0.61 (0.73)	1.12***	0.52**
	Oct–Dec 2021	12.43 (1.37)	− 0.65 (0.73)	0.59 ^†^	0.16
	Jan–Mar 2022	11.84 (1.26)	− 0.41 (0.69)	0.37	− 0.01
	Apr–Jun 2022	11.44 (1.29)	− 0.60 (0.75)	0.45	0.01

*N* of counties = 56, *N* of observations = 1008; Months are abbreviated; Numbers in parentheses are clustered standard errors;

a Fertility rate in a quarter (e.g., April–June 2018)–the 2014–2017 average in the corresponding quarter (e.g., April–June);

b Republican-leaning counties’ fertility rate–Democraticleaning counties’ fertility rate;

c Republican-leaning counties’ excess fertility rate–Democratic-leaning counties’ excess fertility rate.

† *p* < .10,

* *p* < .05,

** *p* < .01,

*** *p* < .001 (two-tailed tests)

**Table 3:** Quarterly change in excess fertility rates before and during the COVID-19 pandemic by 2020 County partisanship

Time period	Birth quarter	Trump majority^a^		Trump supermajority^b^		Trump % vote share	
		Model 1A	Model 2A	Model 1B	Model 2B	Model 1C	Model 2C
Pre-pandemic birth and conception	Apr–Jun 2018	0.106 (0.156)	0.120 (0.188)	0.095 (0.213)	0.097 (0.217)	0.005 (0.008)	0.006 (0.009)
	Jul–Sep 2018	0.147 (0.152)	0.069 (0.180)	0.322 (0.249)	0.275 (0.259)	0.006 (0.009)	0.002 (0.011)
	Oct–Dec 2018	− 0.107 (0.183)	− 0.077 (0.199)	− 0.351 (0.313)	− 0.307 (0.311)	− 0.014 (0.009)	− 0.014 (0.010)
	Jan–Mar 2019	0.168 (0.190)	0.200 (0.214)	− 0.162 (0.263)	− 0.129 (0.261)	0.002 (0.009)	0.003 (0.010)
	Apr–Jun 2019	0.038 (0.156)	0.043 (0.183)	− 0.181 (0.244)	− 0.153 (0.255)	− 0.008 (0.009)	− 0.008 (0.010)
	Jul–Sep 2019	0.001 (0.169)	− 0.026 (0.191)	0.052 (0.260)	0.094 (0.232)	0.003 (0.009)	0.002 (0.010)
	Oct–Dec 2019	− 0.060 (0.202)	− 0.028 (0.192)	− 0.254 (0.249)	− 0.218 (0.253)	− 0.008 (0.008)	− 0.006 (0.009)
Pandemic birth, but not conception	Jan–Mar 2020	− 0.230 (0.140)	− 0.202 (0.167)	− 0.222 (0.226)	− 0.175 (0.236)	− 0.012 (0.008)	− 0.010 (0.008)
	Apr–Jun 2020	0.229 (0.169)	0.265 (0.182)	0.206 (0.221)	0.258 (0.221)	0.007 (0.009)	0.009 (0.009)
	Jul–Sep 2020	0.382^†^ (0.194)	0.399^†^ (0.202)	0.172 (0.256)	0.212 (0.252)	0.011 (0.009)	0.013 (0.010)
Pandemic birth and conception	Oct–Dec 2020	0.578* (0.260)	0.525* (0.239)	0.307 (0.326)	0.312 (0.305)	0.022^†^ (0.011)	0.023^†^ (0.012)
	Jan–Mar 2021	0.794*** (0.213)	0.824*** (0.236)	0.538* (0.237)	0.623* (0.284)	0.030** (0.009)	0.035** (0.012)
	Apr–Jun 2021	0.396^†^ (0.203)	0.237 (0.225)	0.263 (0.226)	0.130 (0.232)	0.016 (0.011)	0.009 (0.011)
	Jul–Sep 2021	0.573* (0.237)	0.497 (0.333)	0.322 (0.309)	0.189 (0.333)	0.022^†^ (0.011)	0.019 (0.014)
	Oct–Dec 2021	0.213 (0.232)	0.218 (0.342)	0.079 (0.335)	0.171 (0.388)	− 0.001 (0.010)	− 0.004 (0.014)
	Jan–Mar 2022	0.045 (0.230)	0.259 (0.283)	− 0.238 (0.322)	0.004 (0.338)	− 0.007 (0.011)	0.002 (0.012)
	Apr–Jun 2022	0.065 (0.259)	− 0.009 (0.331)	− 0.192 (0.270)	− 0.158 (0.328)	− 0.007 (0.011)	− 0.012 (0.013)

N of counties = 56, N of observations = 1008; Months are abbreviated; Numbers in parentheses are clustered standard errors; All models include time and county fixed effects and Model 2 also includes controls for county-level race/ethnicity and unemployment changes (see Online Supplement Table S3 for full model estimates).

a Trump majority denotes ≥ 50% 2020 Trump vote share.

b Trump supermajority denotes ≥ 60% 2020 Trump vote share.

† *p* < .10,

* *p* < .05,

** *p* < .01,

*** *p* < .001 (two-tailed tests).

**Table 4:** Quarterly change in excess fertility rates before and during the COVID-19 pandemic by 2020 county partisanship, White women

Time period	Birth quarter	Trump majority^a^		Trump supermajority^b^		Trump % vote share	
		Model 1A	Model 2A	Model 1B	Model 2B	Model 1C	Model 2C
Pre-pandemic birth and conception	Apr–Jun 2018	0.174 (0.139)	0.122 (0.178)	0.131 (0.222)	0.107 (0.228)	0.004 (0.007)	0.001 (0.008)
	Jul–Sep 2018	0.269^†^ (0.156)	0.115 (0.188)	0.380 (0.276)	0.297 (0.290)	0.010 (0.008)	0.004 (0.010)
	Oct–Dec 2018	0.004 (0.201)	− 0.058 (0.217)	− 0.322 (0.338)	− 0.346 (0.331)	− 0.015 (0.010)	− 0.019^†^ (0.011)
	Jan–Mar 2019	0.220 (0.182)	0.173 (0.207)	**0.091** (0.275)	**0.070** (0.276)	0.002 (0.010)	0.000 (0.010)
	Apr–Jun 2019	0.156 (0.199)	0.121 (0.220)	− 0.177 (0.293)	− 0.192 (0.304)	− 0.009 (0.011)	− 0.011 (0.012)
	Jul–Sep 2019	0.149 (0.220)	0.033 (0.196)	0.182 (0.346)	0.170 (0.298)	0.003 (0.010)	− 0.002 (0.010)
	Oct–Dec 2019	− 0.024 (0.212)	− 0.069 (0.213)	− 0.265 (0.276)	− 0.301 (0.287)	− 0.014 (0.009)	− 0.016 (0.010)
Pandemic birth, but not conception	Jan–Mar 2020	− 0.247 (0.161)	− 0.296 (0.187)	− 0.070 (0.250)	− 0.096 (0.262)	− 0.014^†^ (0.007)	− 0.016^†^ (0.009)
	Apr–Jun 2020	0.384* (0.170)	0.346^†^ (0.184)	0.319 (0.239)	0.298 (0.239)	0.010 (0.009)	0.009 (0.009)
	Jul–Sep 2020	0.634* (0.258)	0.561* (0.212)	0.354 (0.319)	0.309 (0.291)	0.013 (0.011)	0.012 (0.010)
Pandemic birth and conception	Oct–Dec 2020	0.741* (0.289)	0.568* (0.216)	*0.484* (0.370)	0.368 (0.339)	0.023^†^ (0.012)	0.020^†^ (0.011)
	Jan–Mar 2021	0.958*** (0.176)	0.854*** (0.234)	0.885*** (0.232)	0.774* (0.309)	0.037*** (0.010)	0.032* (0.014)
	Apr–Jun 2021	0.536* (0.204)	0.079 (0.272)	0.537* (0.229)	0.156 (0.287)	**0.019** (0.012)	− 0.004 (0.015)
	Jul–Sep 2021	0.677** (0.208)	0.564 (0.373)	0.485 (0.306)	0.295 (0.345)	0.025* (0.011)	0.020 (0.015)
	Oct–Dec 2021	0.319 (0.242)	− 0.097 (0.309)	**0.337** (0.369)	0.110 (0.420)	**0.008** (0.013)	− 0.012 (0.014)
	Jan–Mar 2022	− 0.059 (0.236)	− 0.036 (0.251)	− 0.183 (0.317)	− 0.071 (0.313)	− 0.011 (0.013)	− 0.010 (0.013)
	Apr–Jun 2022	− 0.022 (0.252)	− 0.052 (0.249)	− 0.169 (0.306)	− 0.122 (0.299)	− 0.010 (0.014)	− 0.012 (0.014)

N of counties = 55, N of observations = 990; Months are abbreviated; Numbers in parentheses are clustered standard errors; All models include time and county fixed effects and Model 2 also includes controls for county-level race/ethnicity and unemployment changes (see Online Supplement Table S4 for full model estimates).

a: Trump majority denotes ≥ 50% 2020 Trump vote share.

b: Trump supermajority denotes ≥ 60% 2020 Trump vote share.

† *p* < .10,

* *p* < .05,

** *p* < .01,

*** *p* < .001 (two-tailed tests); coefficients in **bold** or *italics* differ from the corresponding coefficient for non-White women in Table 5 at *p* < .05 and *p* < .10, respectively.

**Table 5:** Quarterly change in excess fertility rates before and during the COVID-19 pandemic by 2020 county partisanship, Non-White women

Time period	Birth quarter	Trump majority^a^		Trump supermajority^b^		Trump % vote share	
		Model 1A	Model 2 A	Model 1B	Model 2B	Model 1C	Model 2C
Pre-pandemic birth and conception	Apr–Jun 2018	− 0.080 (0.459)	0.089 (0.463)	− 0.059 (0.701)	0.005 (0.667)	0.005 (0.022)	0.011 (0.021)
	Jul–Sep 2018	− 0.280 (0.389)	− 0.277 (0.416)	0.009 (0.608)	− 0.001 (0.633)	− 0.012 (0.021)	− 0.015 (0.023)
	Oct–Dec 2018	− 0.560 (0.389)	− 0.288 (0.347)	− 0.505 (0.659)	− 0.326 (0.632)	− 0.024 (0.021)	− 0.011 (0.021)
	Jan–Mar 2019	− 0.079 (0.463)	0.192 (0.498)	− 1.243* (0.559)	− 1.123* (0.544)	− 0.009 (0.022)	0.002 (0.023)
	Apr–Jun 2019	− 0.412 (0.358)	− 0.396 (0.368)	− 0.377 (0.575)	− 0.341 (0.571)	− 0.013 (0.019)	− 0.011 (0.019)
	Jul–Sep 2019	− 0.549 (0.611)	− 0.414 (0.616)	− 0.475 (0.969)	− 0.376 (0.958)	− 0.010 (0.031)	− 0.004 (0.030)
	Oct–Dec 2019	− 0.285 (0.475)	− 0.114 (0.471)	− 0.525 (0.790)	− 0.419 (0.797)	− 0.004 (0.023)	0.005 (0.023)
Pandemic birth, but not conception	Jan–Mar 2020	− 0.187 (0.435)	− 0.016 (0.444)	− 0.780 (0.734)	− 0.676 (0.725)	− 0.013 (0.021)	− 0.004 (0.022)
	Apr–Jun 2020	− 0.187 (0.451)	− 0.022 (0.445)	− 0.167 (0.712)	− 0.049 (0.710)	− 0.003 (0.023)	0.005 (0.022)
	Jul–Sep 2020	− 0.063 (0.512)	0.096 (0.511)	− 0.329 (0.657)	− 0.219 (0.646)	0.001 (0.024)	0.009 (0.023)
Pandemic birth and conception	Oct–Dec 2020	0.118 (0.454)	0.226 (0.458)	*–0.536* (0.515)	− 0.442 (0.507)	0.010 (0.020)	0.018 (0.021)
	Jan–Mar 2021	− **0.012** (0.462)	*0.216* (0.468)	− **1.062**^†^ (0.535)	− **0.844** (0.567)	− **0.012** (0.018)	0.003 (0.021)
	Apr–Jun 2021	− **0.462** (0.487)	− 0.114 (0.560)	− **1.088** (0.786)	− 0.846 (0.846)	− 0.016 (0.024)	0.005 (0.029)
	Jul–Sep 2021	− 0.057 (0.570)	− 0.078 (0.696)	− 0.301 (0.639)	− 0.299 (0.719)	− 0.002 (0.023)	− 0.003 (0.027)
	Oct–Dec 2021	− 0.329 (0.487)	0.344 (0.681)	− **1.209**^†^ (0.652)	− 0.827 (0.705)	− **0.036**^†^ (0.019)	− 0.022 (0.027)
	Jan–Mar 2022	0.272 (0.514)	0.989 (0.702)	− 0.416 (0.669)	− 0.016 (0.740)	− 0.006 (0.020)	0.020 (0.027)
	Apr–Jun 2022	− 0.390 (0.456)	− 0.102 (0.631)	− 0.876 (0.605)	− 0.585 (0.635)	− 0.028 (0.020)	− 0.017 (0.026)

N of counties = 56, N of observations = 1008; Months are abbreviated; Numbers in parentheses are clustered standard errors; All models include time and county fixed effects and Model 2 also includes controls for county-level race/ethnicity and unemployment changes (see Online Supplement Table S5 for full model estimates).

a: Trump majority denotes ≥ 50% 2020 Trump vote share.

b: Trump supermajority denotes ≥ 60% 2020 Trump vote share.

† *p* < .10,

* *p* < .05,

** *p* < .01,

*** *p* < .001 (two-tailed tests); coefficients in **bold** or *italics* differ from the corresponding coefficient for White women in Table 4 at *p* < .05 and *p* < .10, respectively.

## Data Availability

The data was aggregated to the county-level and publicly available and, thus, were exempt from IRB approval. Data is available at https://www.flhealthcharts.gov/.

## Footnotes

1 If instead fertility changes were similar across racial groups, this is suggestive that something about being in a Republican- or Democratic-leaning county matters (e.g., county-level policies, like school closures, may differ) rather than the politically informed views of women themselves and/or of their relevant social others (albeit other time varying county characteristic could result in similarity by race, such as offsetting changes in population composition; other potential reasons for racial differences are discussed in the conclusion).

2 Publicly available quarterly data on fertility stratified by race and ethnicity were not available when this article was written.

3 Like Dahl et al. (2022) we used quarterly data because monthly birth counts were not available by race.

4 Compared to included counties, excluded ones did not significantly differ in terms of any measured variable, but had marginally-higher Trump vote shares (*p* < .10). Models that included all counties were substantively similar to our primary findings, but the results for non-White women’s fertility were unstable as excluded counties had no or few non-White births (not shown).

5 We used the 10th percentile of non-White births among counties that had at least one non-White birth (including the six counties that had quarters with zero non-White births would have made the 10th percentile cutoff zero).

6 Based on yearly data that includes details on fertility stratified by race and ethnicity, we found the vast majority of non-White births are born to Black women. We further assume that most Hispanic women are counted as White, given that the majority of Hispanic Floridians identify as White (Data USA: Florida, 2024). Also, among Hispanic voters, the vote was almost evenly split between Biden and Trump in 2020 (Barreto & Gutierrez 2022), suggesting that White Hispanic Floridians voting resembles that of White non-Hispanic Floridians.

7 Nearly all counties without a Republican voting majority had a Democratic one, as third-party vote shares were low (Pinellas County was the only county where neither party exceeded 50% of the vote).

8 Results without fixed effects were similar because excess fertility reflects within-county change. Like Dahl et al. (2022), we show fixed effects models to be conservative and ensure results are unbiased by time invariant factors.

9 Racial composition was a significant predictor of excess fertility (fertility declined less as Hispanic populations grew, see Online Supplement Table S3), but it did not explain partisan gaps or alter over-time trends in excess fertility

10 For White women, majority Trump vote share counties had higher excess fertility than non-majority Trump counties in April–September 2020, when children were born during the pandemic but conceived before the pandemic; nevertheless, significant positive partisan gaps were only found for one of three Trump support measures in this period. Because the vast majority of Hispanic Floridians identify as White, perhaps COVID-19 travel restrictions prevented foreign-born pregnant Hispanic women from giving birth in Florida which may have depressed White women’s fertility rates more in the Democratic-leaning counties that most Hispanic immigrants would move to.

11 Partisan gaps were larger for the continuous and majority Trump measures in adjusted and unadjusted models.

12 In unadjusted models, the continuous measure did not predict excess fertility for marital (*b* = 0.01) or non-marital (*b* = − 0.02) births, but the coefficients differed (*p* < .05). In adjusted models, the coefficients reduced and no longer statistically differed.

13 We interacted education with time because we were interested in how education’s associations varied before and during the COVID-19 pandemic. In fixed effects models, we can only measure variation over time for time invariant factors.

14 Models such as SARIMA with Exogenous Variables can incorporate external factors like political lean, but we did not use this approach due to the limited number of quarterly observations. Adding complexity to these models can lead to overfitting and unreliable estimates. Thus, we could not directly test for county-level partisan differences.

15 When examining all counties we see declines in fertility of 6% lower than expected in July–September 2020, 7% in October–December 2020, 6% in January–March 2021, and a return to expected after. This is consistent with Nobles et al.’s (2024) findings in California.
