# Supplementary material for: Partisan Divergence in Fertility Change Before and During the COVID-19 Pandemic in Florida
Source: Popul Res Policy Rev. Author manuscript; Available in PMC 2026 Apr 24. (PMC13105308; doi:10.1007/s11113-025-09972-0)
Supplement: Supplement Materials [file NIHMS2156393-supplement-Supplement_Materials.docx]

ONLINE SUPPLEMENT

Partisan Divergence in Fertility Change Before and During the COVID-19 Pandemic in Florida

| Table S1. Quarterly Average Fertility Rates Before COVID-19 Pandemic (2014–2017) Used to Calculate Excess Fertility | | | | | | | |
| --- | --- | --- | --- | --- | --- | --- | --- |
|  |  |  |  | County-Level 2020 Partisanship | |  |  |
| Birth Quarter |  | Overall |  | Republican-Leaning ^a^ | Democratic-Leaning ^b^ |  | Partisan Gap ^c^ |
| Jan­Mar |  | 12.24 |  | 12.48 | 12.07 |  | 0.41 |
|  |  | (1.13) |  | (1.28) | (1.02) |  |  |
| Apr­Jun |  | 12.04 |  | 12.32 | 11.85 |  | 0.47 |
|  |  | (1.07) |  | (1.15) | (1.00) |  |  |
| Jul­Sep |  | 13.35 |  | 13.73 | 13.09 |  | 0.64^†^ |
|  |  | (1.27) |  | (1.36) | (1.18) |  |  |
| Oct­Dec |  | 13.08 |  | 13.35 | 12.88 |  | 0.36 |
|  |  | (1.31) |  | (1.49) | (1.16) |  |  |
| *Note. N* of counties = 56, *N* of observations = 1,008; Months are abbreviated; Numbers in parentheses are clustered standard errors. ^:a^ Trump 2020 vote share ≥ 50%; ^b^ Trump 2020 vote share < 50%; ^c^ Republican-leaning areas’ fertility rate - Democratic-leaning areas’ fertility rate. ^†^ *p* < .10 (two-tailed tests). | | | | | | | |

| **Table S2.** **Quarterly Fertility Rate and Partisan Gap, by Conception and Birth Time Period Before and During the COVID-19 Pandemic** | | | | | | | | |
| --- | --- | --- | --- | --- | --- | --- | --- | --- |
|  |  |  |  |  | County-Level 2020 Partisanship | |  |  |
|  | Birth Quarter |  | Overall |  | Republican-Leaning ^a^ | Democratic-Leaning ^b^ |  | Partisan Gap ^c^ |
| Pre-Pandemic Conception and Birth | Jan–Mar 2018 |  | 11.99 |  | 12.20 | 11.85 |  | 0.35 |
|  |  |  | (1.14) |  | (1.34) | (0.99) |  |  |
|  | Apr–Jun 2018 |  | 11.57 |  | 11.88 | 11.36 |  | 0.52 |
|  |  |  | (1.25) |  | (1.39) | (1.14) |  |  |
|  | Jul–Sep 2018 |  | 12.71 |  | 13.13 | 12.41 |  | 0.73* |
|  |  |  | (1.36) |  | (1.45) | (1.24) |  |  |
|  | Oct–Dec 2018 |  | 12.64 |  | 12.81 | 12.51 |  | 0.3 |
|  |  |  | (1.25) |  | (1.36) | (1.20) |  |  |
|  | Jan–Mar 2019 |  | 11.65 |  | 11.96 | 11.44 |  | 0.52 |
|  |  |  | (1.24) |  | (1.44) | (1.06) |  |  |
|  | Apr–Jun 2019 |  | 11.43 |  | 11.70 | 11.25 |  | 0.45 |
|  |  |  | (1.06) |  | (1.07) | (1.05) |  |  |
|  | Jul–Sep 2019 |  | 12.74 |  | 13.07 | 12.50 |  | 0.58^†^ |
|  |  |  | (1.44) |  | (1.65) | (1.27) |  |  |
|  | Oct–Dec 2019 |  | 12.37 |  | 12.57 | 12.22 |  | 0.34 |
|  |  |  | (1.34) |  | (1.54) | (1.21) |  |  |
| Pandemic Birth, but Not Conception |  |  |  |  |  |  |  |  |
|  | Jan–Mar 2020 |  | 11.38 |  | 11.45 | 11.33 |  | 0.12 |
|  |  |  | (1.09) |  | (1.24) | (1.00) |  |  |
|  | Apr–Jun 2020 |  | 10.99 |  | 11.36 | 10.73 |  | 0.63^†^ |
|  |  |  | (1.19) |  | (1.36) | (1.01) |  |  |
|  | Jul–Sep 2020 |  | 11.83 |  | 12.39 | 11.44 |  | 0.95** |
|  |  |  | (1.28) |  | (1.32) | (1.13) |  |  |
| Pandemic Conception and Birth |  |  |  |  |  |  |  |  |
|  | Oct–Dec 2020 |  | 11.62 |  | 12.18 | 11.21 |  | 0.97** |
|  |  |  | (1.42) |  | (1.54) | (1.22) |  |  |
|  | Jan–Mar 2021 |  | 10.94 |  | 11.60 | 10.47 |  | 1.13*** |
|  |  |  | (1.29) |  | (1.41) | (0.98) |  |  |
|  | Apr–Jun 2021 |  | 11.2 |  | 11.66 | 10.87 |  | 0.79* |
|  |  |  | (1.26) |  | (1.24) | (1.20) |  |  |
|  | Jul–Sep 2021 |  | 12.75 |  | 13.41 | 12.28 |  | 1.12*** |
|  |  |  | (1.43) |  | (1.50) | (1.22) |  |  |
|  | Oct–Dec 2021 |  | 12.43 |  | 12.77 | 12.18 |  | 0.59^†^ |
|  |  |  | (1.37) |  | (1.54) | (1.21) |  |  |
|  | Jan–Mar 2022 |  | 11.84 |  | 12.05 | 11.68 |  | 0.37 |
|  |  |  | (1.26) |  | (1.48) | (1.08) |  |  |
|  | Apr–Jun 2022 |  | 11.44 |  | 11.70 | 11.25 |  | 0.45 |
|  |  |  | (1.29) |  | (1.41) | (1.20) |  |  |
| *Note*. *N* of counties = 56, *N* of observations = 1,008; Months are abbreviated; Numbers in parentheses are clustered standard errors; ^a^ Trump 2020 vote share ≥ 50% (Republican-Leaning Area); ^b^ Trump 2020 vote share < 50% (Democratic-Leaning Area); ^c^ Republican-leaning areas’ fertility rate - Democratic-leaning areas’ fertility rate. † *p* < .10, * *p* < .05, ** *p* < .01, *** *p* < .001 (two-tailed tests). | | | | | | | | |

| Table S3. Quarterly Change in Excess Fertility Rates by 2020 Trump Vote Share Before and During the COVID-19 Pandemic | | | | | | | | | |  |
| --- | --- | --- | --- | --- | --- | --- | --- | --- | --- | --- |
|  |  | Trump Majority ^b^ | |  | Trump Supermajority ^c^ | |  | Trump % Vote Share | |  |
|  | Birth Quarter ^a^ | Model 1A | Model 2A |  | Model 1B | Model 2B |  | Model 1C | Model 2C |  |
| Pre-Pandemic Birth  and Conception | Apr–Jun 2018 | -0.261* | -0.142 |  | -0.234* | -0.237 |  | -0.478 | -0.416 |  |
|  |  | (0.115) | (0.572) |  | (0.090) | (0.536) |  | (0.420) | (0.559) |  |
|  | Jul–Sep 2018 | -0.458*** | -0.999^†^ |  | -0.453*** | -0.996^†^ |  | -0.713 | -1.136^†^ |  |
|  |  | (0.101) | (0.553) |  | (0.075) | (0.503) |  | (0.483) | (0.586) |  |
|  | Oct–Dec 2018 | -0.147 | -0.065 |  | -0.130 | -0.124 |  | 0.536 | 0.330 |  |
|  |  | (0.115) | (0.518) |  | (0.088) | (0.481) |  | (0.459) | (0.571) |  |
|  | Jan–Mar 2019 | -0.409*** | -0.301 |  | -0.312** | -0.449 |  | -0.437 | -0.553 |  |
|  |  | (0.114) | (0.512) |  | (0.108) | (0.443) |  | (0.451) | (0.565) |  |
|  | Apr–Jun 2019 | -0.375** | -0.517 |  | -0.328*** | -0.589 |  | 0.028 | -0.323 |  |
|  |  | (0.115) | (0.582) |  | (0.084) | (0.570) |  | (0.473) | (0.586) |  |
|  | Jul–Sep 2019 | -0.370*** | -1.321** |  | -0.379*** | -1.471** |  | -0.525 | -1.516** |  |
|  |  | (0.099) | (0.453) |  | (0.084) | (0.480) |  | (0.469) | (0.498) |  |
|  | Oct–Dec 2019 | -0.435** | 0.045 |  | -0.417*** | -0.095 |  | -0.033 | 0.207 |  |
|  |  | (0.146) | (0.495) |  | (0.116) | (0.441) |  | (0.443) | (0.553) |  |
| Pandemic Birth, but Not Conception |  |  |  |  |  |  |  |  |  |  |
|  | Jan–Mar 2020 | -0.519*** | -0.521 |  | -0.576*** | -0.699 |  | -0.031 | -0.228 |  |
|  |  | (0.080) | (0.456) |  | (0.065) | (0.487) |  | (0.381) | (0.535) |  |
|  | Apr–Jun 2020 | -0.897*** | -0.578 |  | -0.838*** | -0.731^†^ |  | -1.151* | -1.036* |  |
|  |  | (0.124) | (0.445) |  | (0.102) | (0.411) |  | (0.454) | (0.515) |  |
|  | Jul–Sep 2020 | -1.435*** | -0.987* |  | -1.307*** | -1.069* |  | -1.811*** | -1.545** |  |
|  |  | (0.149) | (0.491) |  | (0.132) | (0.459) |  | (0.489) | (0.499) |  |
| Pandemic Birth  and Conception |  |  |  |  |  |  |  |  |  |  |
|  | Oct–Dec 2020 | -1.449*** | -1.112* |  | -1.262*** | -1.184** |  | -2.299*** | -2.142*** |  |
|  |  | (0.207) | (0.493) |  | (0.184) | (0.442) |  | (0.612) | (0.609) |  |
|  | Jan–Mar 2021 | -1.383*** | -1.462* |  | -1.146*** | -1.516* |  | -2.545*** | -3.158** |  |
|  |  | (0.155) | (0.569) |  | (0.170) | (0.599) |  | (0.484) | (0.921) |  |
|  | Apr–Jun 2021 | -0.755*** | -0.531 |  | -0.637*** | -0.731 |  | -1.373* | -1.056 |  |
|  |  | (0.162) | (0.517) |  | (0.132) | (0.445) |  | (0.597) | (0.650) |  |
|  | Jul–Sep 2021 | -0.592*** | -0.624 |  | -0.410** | -0.797^†^ |  | -1.485* | -1.549* |  |
|  |  | (0.156) | (0.474) |  | (0.151) | (0.420) |  | (0.582) | (0.616) |  |
|  | Oct–Dec 2021 | -0.485** | -0.539 |  | -0.411** | -0.768^†^ |  | -0.369 | -0.453 |  |
|  |  | (0.161) | (0.505) |  | (0.130) | (0.445) |  | (0.515) | (0.698) |  |
|  | Jan–Mar 2022 | -0.176 | -0.047 |  | -0.117 | -0.285 |  | 0.214 | -0.270 | |
|  |  | (0.134) | (0.458) |  | (0.118) | (0.409) |  | (0.561) | (0.564) | |
|  | Apr–Jun 2022 | -0.378† | -0.587 |  | -0.319* | -0.787 |  | -0.009 | -0.291 | |
|  |  | (0.210) | (0.518) |  | (0.158) | (0.472) |  | (0.598) | (0.608) | |
|  | | | | | | | | | | |
| ***(Table S3. Continued)*** | | | | | | | | | | |
| Quarter $\times$ Trump Support (i.e., Partisan Excess Fertility Gap) | | | | | | | | | | |
| Pre-Pandemic Birth  and Conception | Apr–Jun 2018 $\times$  Trump | 0.106 | 0.120 |  | 0.095 | 0.097 |  | 0.005 | 0.006 | |
|  |  | (0.156) | (0.188) |  | (0.213) | (0.217) |  | (0.008) | (0.009) | |
|  | Jul–Sep 2018 $\times$  Trump | 0.147 | 0.069 |  | 0.322 | 0.275 |  | 0.006 | 0.002 | |
|  |  | (0.152) | (0.180) |  | (0.249) | (0.259) |  | (0.009) | (0.011) | |
|  | Oct–Dec 2018 $\times$  Trump | -0.107 | -0.077 |  | -0.351 | -0.307 |  | -0.014 | -0.014 | |
|  |  | (0.183) | (0.199) |  | (0.313) | (0.311) |  | (0.009) | (0.010) | |
|  | Jan–Mar 2019 $\times$  Trump | 0.168 | 0.200 |  | -0.162 | -0.129 |  | 0.002 | 0.003 | |
|  |  | (0.190) | (0.214) |  | (0.263) | (0.261) |  | (0.009) | (0.010) | |
|  | Apr–Jun 2019 $\times$  Trump | 0.038 | 0.043 |  | -0.181 | -0.153 |  | -0.008 | -0.008 | |
|  |  | (0.156) | (0.183) |  | (0.244) | (0.255) |  | (0.009) | (0.010) | |
|  | Jul–Sep 2019 $\times$  Trump | 0.001 | -0.026 |  | 0.052 | 0.094 |  | 0.003 | 0.002 | |
|  |  | (0.169) | (0.191) |  | (0.260) | (0.232) |  | (0.009) | (0.010) | |
|  | Oct–Dec 2019 $\times$  Trump | -0.060 | -0.028 |  | -0.254 | -0.218 |  | -0.008 | -0.006 | |
|  |  | (0.202) | (0.192) |  | (0.249) | (0.253) |  | (0.008) | (0.009) | |
| Pandemic Birth,  but Not Conception |  |  |  |  |  |  |  |  |  | |
|  | Jan–Mar 2020 $\times$  Trump | -0.230 | -0.202 |  | -0.222 | -0.175 |  | -0.012 | -0.010 | |
|  |  | (0.140) | (0.167) |  | (0.226) | (0.236) |  | (0.008) | (0.008) | |
|  | Apr–Jun 2020 $\times$  Trump | 0.229 | 0.265 |  | 0.206 | 0.258 |  | 0.007 | 0.009 | |
|  |  | (0.169) | (0.182) |  | (0.221) | (0.221) |  | (0.009) | (0.009) | |
|  | Jul–Sep 2020 $\times$  Trump | 0.382^†^ | 0.399^†^ |  | 0.172 | 0.212 |  | 0.011 | 0.013 | |
|  |  | (0.194) | (0.202) |  | (0.256) | (0.252) |  | (0.009) | (0.010) | |
| Pandemic Birth  and Conception |  |  |  |  |  |  |  |  |  | |
|  | Oct–Dec 2020 $\times$  Trump | 0.578* | 0.525* |  | 0.307 | 0.312 |  | 0.022^†^ | 0.023^†^ | |
|  |  | (0.260) | (0.239) |  | (0.326) | (0.305) |  | (0.011) | (0.012) | |
|  | Jan–Mar 2021 $\times$  Trump | 0.794*** | 0.824*** |  | 0.538* | 0.623* |  | 0.030** | 0.035** | |
|  |  | (0.213) | (0.236) |  | (0.237) | (0.284) |  | (0.009) | (0.012) | |
|  | Apr–Jun 2021 $\times$  Trump | 0.396^†^ | 0.237 |  | 0.263 | 0.130 |  | 0.016 | 0.009 | |
|  |  | (0.203) | (0.225) |  | (0.226) | (0.232) |  | (0.011) | (0.011) | |
|  | Jul–Sep 2021 $\times$  Trump | 0.573* | 0.497 |  | 0.322 | 0.189 |  | 0.022^†^ | 0.019 | |
|  |  | (0.237) | (0.333) |  | (0.309) | (0.333) |  | (0.011) | (0.014) | |
|  | Oct–Dec 2021 $\times$  Trump | 0.213 | 0.218 |  | 0.079 | 0.171 |  | -0.001 | -0.004 | |
|  |  | (0.232) | (0.342) |  | (0.335) | (0.388) |  | (0.010) | (0.014) | |
|  | Jan–Mar 2022 $\times$  Trump | 0.045 | 0.259 |  | -0.238 | 0.004 |  | -0.007 | 0.002 | |
|  |  | (0.230) | (0.283) |  | (0.322) | (0.338) |  | (0.011) | (0.012) | |
|  | Apr–Jun 2022 $\times$  Trump | 0.065 | -0.009 |  | -0.192 | -0.158 |  | -0.007 | -0.012 | |
|  |  | (0.259) | (0.331) |  | (0.270) | (0.328) |  | (0.011) | (0.013) | |
|  |  |  |  |  |  |  |  |  |  | |
|  | Excess Change in  Hispanic Pop. % | — | 0.136^†^ |  | — | 0.150^†^ |  | — | 0.156* | |
|  |  |  | (0.068) |  |  | (0.089) |  |  | (0.076) | |
|  | Excess Change in  Black Pop. % | — | -0.093 |  | — | -0.079 |  | — | -0.082 | |
|  |  |  | (0.121) |  |  | (0.129) |  |  | (0.118) | |
|  | Excess Change in  Unemployment | — | -0.081 |  | — | 0.059 |  | — | -0.015 | |
|  |  |  | (0.256) |  |  | (0.215) |  |  | (0.245) | |
|  |  | — | 0.136^†^ |  | — | 0.150^†^ |  | — | 0.156* | |
| ***(Table S3. Continued)*** | | | | | | | | | | |
| Quarter $\times$ Excess Unemployment | | | | | | | | | | |
| Pre-Pandemic Birth  and Conception | Apr–Jun 2018 $\times$  Unemp. | — | 0.065 |  | — | -0.000 |  | — | 0.042 | |
|  |  |  | (0.323) |  |  | (0.281) |  |  | (0.307) | |
|  | Jul–Sep 2018 $\times$  Unemp. | — | -0.331 |  | — | -0.307 |  | — | -0.351 | |
|  |  |  | (0.316) |  |  | (0.275) |  |  | (0.316) | |
|  | Oct–Dec 2018 $\times$  Unemp. | — | 0.083 |  | — | 0.062 |  | — | -0.063 | |
|  |  |  | (0.299) |  |  | (0.265) |  |  | (0.283) | |
|  | Jan–Mar 2019 $\times$  Unemp. | — | 0.093 |  | — | -0.038 |  | — | 0.011 | |
|  |  |  | (0.255) |  |  | (0.198) |  |  | (0.228) | |
|  | Apr–Jun 2019 $\times$  Unemp. | — | -0.008 |  | — | -0.086 |  | — | -0.103 | |
|  |  |  | (0.266) |  |  | (0.237) |  |  | (0.261) | |
|  | Jul–Sep 2019 $\times$  Unemp. | — | -0.377 |  | — | -0.450* |  | — | -0.410^†^ | |
|  |  |  | (0.234) |  |  | (0.216) |  |  | (0.240) | |
|  | Oct–Dec 2019 $\times$  Unemp. | — | 0.301 |  | — | 0.218 |  | — | 0.232 | |
|  |  |  | (0.269) |  |  | (0.225) |  |  | (0.245) | |
| Pandemic Birth,  but Not Conception |  |  |  |  |  |  |  |  |  | |
|  | Jan–Mar 2020 $\times$  Unemp. | — | 0.067 |  | — | -0.018 |  | — | 0.005 | |
|  |  |  | (0.239) |  |  | (0.228) |  |  | (0.234) | |
|  | Apr–Jun 2020 $\times$  Unemp. | — | 0.182 |  | — | 0.065 |  | — | 0.129 | |
|  |  |  | (0.246) |  |  | (0.206) |  |  | (0.233) | |
|  | Jul–Sep 2020 $\times$  Unemp. | — | 0.233 |  | — | 0.119 |  | — | 0.190 | |
|  |  |  | (0.256) |  |  | (0.216) |  |  | (0.245) | |
| Pandemic Birth  and Conception |  |  |  |  |  |  |  |  |  | |
|  | Oct–Dec 2020 $\times$  Unemp. | — | 0.273 |  | — | 0.163 |  | — | 0.236 | |
|  |  |  | (0.258) |  |  | (0.218) |  |  | (0.243) | |
|  | Jan–Mar 2021 $\times$  Unemp. | — | 0.088 |  | — | -0.051 |  | — | 0.041 | |
|  |  |  | (0.254) |  |  | (0.210) |  |  | (0.241) | |
|  | Apr–Jun 2021 $\times$  Unemp. | — | 0.017 |  | — | -0.127 |  | — | -0.043 | |
|  |  |  | (0.251) |  |  | (0.209) |  |  | (0.240) | |
|  | Jul–Sep 2021 $\times$  Unemp. | — | 0.024 |  | — | -0.183 |  | — | -0.048 | |
|  |  |  | (0.271) |  |  | (0.219) |  |  | (0.268) | |
|  | Oct–Dec 2021 $\times$  Unemp. | — | 0.091 |  | — | -0.041 |  | — | -0.058 | |
|  |  |  | (0.301) |  |  | (0.254) |  |  | (0.301) | |
|  | Jan–Mar 2022 $\times$  Unemp. | — | 0.382 |  | — | 0.186 |  | — | 0.246 | |
|  |  |  | (0.283) |  |  | (0.216) |  |  | (0.271) | |
|  | Apr–Jun 2022 $\times$  Unemp. | — | -0.028 |  | — | -0.125 |  | — | -0.175 | |
|  |  |  | (0.327) |  |  | (0.294) |  |  | (0.327) | |
|  |  |  |  |  |  |  |  |  |  | |
|  | Constant | -0.554*** | -0.767^†^ |  | -0.624*** | -0.599 |  | -0.577*** | -0.666 | |
|  |  | (0.097) | (0.449) |  | (0.081) | (0.409) |  | (0.134) | (0.417) | |
|  | R^2^ | 0.448 | 0.465 |  | 0.432 | 0.452 |  | 0.447 | 0.467 | |
| *Note*. *N* of counties = 56, *N* of observations = 1,008; Months are abbreviated; Pop. = population; Unemp. = excess unemployment rate; excess rates calculated based on differences from average for 2014–2017, lagged three quarters (see text for details); Numbers in parentheses are clustered standard errors; All models include county fixed effects. ^a:^ Compared to Jan–Mar 2018; ^b^ Trump majority denotes ≥ 50% 2020 Trump vote share. ^c:^ Trump supermajority denotes ≥ 60% 2020 Trump vote share. ^†^ *p*  < .10, * *p*  < .05, ** *p* < .01, *** *p* < .001 (two-tailed tests). | | | | | | | | | | |

| Table S4. Quarterly Change in Excess Fertility Rates by 2020 Trump Vote Share Before and During the COVID-19 Pandemic, White Women | | | | | | | | | |  |
| --- | --- | --- | --- | --- | --- | --- | --- | --- | --- | --- |
|  |  | Trump Majority ^b^ | |  | Trump Supermajority ^c^ | |  | Trump % Vote Share | |  |
|  | Birth Quarter ^a^ | Model 1A | Model 2A |  | Model 1B | Model 2B |  | Model 1C | Model 2C |  |
| Pre-Pandemic Birth  and Conception | Apr–Jun 2018 | -0.309** | -0.730 |  | -0.260** | -0.824^†^ |  | -0.415 | -0.874^†^ |  |
|  |  | (0.095) | (0.550) |  | (0.076) | (0.488) |  | (0.368) | (0.477) |  |
|  | Jul–Sep 2018 | -0.465*** | -1.614* |  | -0.420*** | -1.649** |  | -0.852* | -1.808** |  |
|  |  | (0.086) | (0.635) |  | (0.072) | (0.576) |  | (0.418) | (0.569) |  |
|  | Oct–Dec 2018 | -0.114 | -0.713 |  | -0.057 | -0.787 |  | 0.625 | -0.178 |  |
|  |  | (0.116) | (0.586) |  | (0.098) | (0.548) |  | (0.500) | (0.617) |  |
|  | Jan–Mar 2019 | -0.408** | -0.854^†^ |  | -0.333** | -0.959* |  | -0.418 | -0.966 |  |
|  |  | (0.122) | (0.483) |  | (0.105) | (0.463) |  | (0.501) | (0.606) |  |
|  | Apr–Jun 2019 | -0.423** | -0.766 |  | -0.328** | -0.886 |  | 0.091 | -0.494 |  |
|  |  | (0.153) | (0.697) |  | (0.115) | (0.708) |  | (0.572) | (0.750) |  |
|  | Jul–Sep 2019 | -0.474*** | -2.176*** |  | -0.444*** | -2.328*** |  | -0.563 | -2.229*** |  |
|  |  | (0.124) | (0.518) |  | (0.105) | (0.543) |  | (0.510) | (0.635) |  |
|  | Oct–Dec 2019 | -0.480*** | -0.602 |  | -0.445*** | -0.723 |  | 0.213 | -0.065 |  |
|  |  | (0.137) | (0.556) |  | (0.116) | (0.508) |  | (0.465) | (0.586) |  |
| Pandemic Birth, but Not Conception |  |  |  |  |  |  |  |  |  |  |
|  | Jan–Mar 2020 | -0.511*** | -1.020* |  | -0.603*** | -1.239* |  | 0.072 | -0.529 |  |
|  |  | (0.094) | (0.508) |  | (0.079) | (0.542) |  | (0.359) | (0.519) |  |
|  | Apr–Jun 2020 | -0.974*** | -1.073* |  | -0.871*** | -1.202* |  | -1.337** | -1.547** |  |
|  |  | (0.123) | (0.486) |  | (0.109) | (0.459) |  | (0.452) | (0.497) |  |
|  | Jul–Sep 2020 | -1.560*** | -1.064^†^ |  | -1.359*** | -1.074^†^ |  | -1.939** | -1.556** |  |
|  |  | (0.219) | (0.550) |  | (0.191) | (0.539) |  | (0.611) | (0.507) |  |
| Pandemic Birth  and Conception |  |  |  |  |  |  |  |  |  |  |
|  | Oct–Dec 2020 | -1.581*** | -1.475** |  | -1.357*** | -1.507*** |  | -2.432*** | -2.395*** |  |
|  |  | (0.237) | (0.482) |  | (0.212) | (0.412) |  | (0.662) | (0.534) |  |
|  | Jan–Mar 2021 | -1.358*** | -1.575* |  | -1.114*** | -1.645* |  | -2.831*** | -3.211** |  |
|  |  | (0.117) | (0.640) |  | (0.146) | (0.679) |  | (0.526) | (1.123) |  |
|  | Apr–Jun 2021 | -0.774*** | -0.714 |  | -0.645*** | -1.025* |  | -1.503* | -0.715 |  |
|  |  | (0.146) | (0.482) |  | (0.127) | (0.439) |  | (0.637) | (0.816) |  |
|  | Jul–Sep 2021 | -0.602*** | -1.110* |  | -0.405** | -1.246** |  | -1.594** | -2.114** |  |
|  |  | (0.117) | (0.483) |  | (0.129) | (0.421) |  | (0.550) | (0.639) |  |
|  | Oct–Dec 2021 | -0.472** | -0.978* |  | -0.399** | -1.238** |  | -0.725 | -0.656 |  |
|  |  | (0.142) | (0.481) |  | (0.126) | (0.419) |  | (0.640) | (0.607) |  |
|  | Jan–Mar 2022 | -0.100 | -0.545 |  | -0.095 | -0.731 |  | 0.445 | -0.275 | |
|  |  | (0.164) | (0.503) |  | (0.131) | (0.467) |  | (0.696) | (0.717) | |
|  | Apr–Jun 2022 | -0.010 | -0.454 |  | 0.008 | -0.595 |  | 0.491 | -0.123 | |
|  |  | (0.192) | (0.570) |  | (0.148) | (0.553) |  | (0.776) | (0.802) | |
|  | | | | | | | | | | |
| ***(Table S4. Continued)*** | | | | | | | | | | |
| Quarter $\times$ Trump Support (i.e., Partisan Excess Fertility Gap) | | | | | | | | | | |
| Pre-Pandemic Birth  and Conception | Apr–Jun 2018 $\times$  Trump | 0.174 | 0.122 |  | 0.131 | 0.107 |  | 0.004 | 0.001 | |
|  |  | (0.139) | (0.178) |  | (0.222) | (0.228) |  | (0.007) | (0.008) | |
|  | Jul–Sep 2018 $\times$  Trump | 0.269^†^ | 0.115 |  | 0.380 | 0.297 |  | 0.010 | 0.004 | |
|  |  | (0.156) | (0.188) |  | (0.276) | (0.290) |  | (0.008) | (0.010) | |
|  | Oct–Dec 2018 $\times$  Trump | 0.004 | -0.058 |  | -0.322 | -0.346 |  | -0.015 | -0.019^†^ | |
|  |  | (0.201) | (0.217) |  | (0.338) | (0.331) |  | (0.010) | (0.011) | |
|  | Jan–Mar 2019 $\times$  Trump | 0.220 | 0.173 |  | 0.091 | 0.070 |  | 0.002 | -0.000 | |
|  |  | (0.182) | (0.207) |  | (0.275) | (0.276) |  | (0.010) | (0.010) | |
|  | Apr–Jun 2019 $\times$  Trump | 0.156 | 0.121 |  | -0.177 | -0.192 |  | -0.009 | -0.011 | |
|  |  | (0.199) | (0.220) |  | (0.293) | (0.304) |  | (0.011) | (0.012) | |
|  | Jul–Sep 2019 $\times$  Trump | 0.149 | 0.033 |  | 0.182 | 0.170 |  | 0.003 | -0.002 | |
|  |  | (0.220) | (0.196) |  | (0.346) | (0.298) |  | (0.010) | (0.010) | |
|  | Oct–Dec 2019 $\times$  Trump | -0.024 | -0.069 |  | -0.265 | -0.301 |  | -0.014 | -0.016 | |
|  |  | (0.212) | (0.213) |  | (0.276) | (0.287) |  | (0.009) | (0.010) | |
| Pandemic Birth,  but Not Conception |  |  |  |  |  |  |  |  |  | |
|  | Jan–Mar 2020 $\times$  Trump | -0.247 | -0.296 |  | -0.070 | -0.096 |  | -0.014^†^ | -0.016^†^ | |
|  |  | (0.161) | (0.187) |  | (0.250) | (0.262) |  | (0.007) | (0.009) | |
|  | Apr–Jun 2020 $\times$  Trump | 0.384* | 0.346^†^ |  | 0.319 | 0.298 |  | 0.010 | 0.009 | |
|  |  | (0.170) | (0.184) |  | (0.239) | (0.239) |  | (0.009) | (0.009) | |
|  | Jul–Sep 2020 $\times$  Trump | 0.634* | 0.561* |  | 0.354 | 0.309 |  | 0.013 | 0.012 | |
|  |  | (0.258) | (0.212) |  | (0.319) | (0.291) |  | (0.011) | (0.010) | |
| Pandemic Birth  and Conception |  |  |  |  |  |  |  |  |  | |
|  | Oct–Dec 2020 $\times$  Trump | 0.741* | 0.568* |  | 0.484 | 0.368 |  | 0.023^†^ | 0.020^†^ | |
|  |  | (0.289) | (0.216) |  | (0.370) | (0.339) |  | (0.012) | (0.011) | |
|  | Jan–Mar 2021 $\times$  Trump | 0.958*** | 0.854*** |  | 0.885*** | 0.774* |  | 0.037*** | 0.032* | |
|  |  | (0.176) | (0.234) |  | (0.232) | (0.309) |  | (0.010) | (0.014) | |
|  | Apr–Jun 2021 $\times$  Trump | 0.536* | 0.079 |  | 0.537* | 0.156 |  | 0.019 | -0.004 | |
|  |  | (0.204) | (0.272) |  | (0.229) | (0.287) |  | (0.012) | (0.015) | |
|  | Jul–Sep 2021 $\times$  Trump | 0.677** | 0.564 |  | 0.485 | 0.295 |  | 0.025* | 0.020 | |
|  |  | (0.208) | (0.373) |  | (0.306) | (0.345) |  | (0.011) | (0.015) | |
|  | Oct–Dec 2021 $\times$  Trump | 0.319 | -0.097 |  | 0.337 | 0.110 |  | 0.008 | -0.012 | |
|  |  | (0.242) | (0.309) |  | (0.369) | (0.420) |  | (0.013) | (0.014) | |
|  | Jan–Mar 2022 $\times$  Trump | -0.059 | -0.036 |  | -0.183 | -0.071 |  | -0.011 | -0.010 | |
|  |  | (0.236) | (0.251) |  | (0.317) | (0.313) |  | (0.013) | (0.013) | |
|  | Apr–Jun 2022 $\times$  Trump | -0.022 | -0.052 |  | -0.169 | -0.122 |  | -0.010 | -0.012 | |
|  |  | (0.252) | (0.249) |  | (0.306) | (0.299) |  | (0.014) | (0.014) | |
|  |  |  |  |  |  |  |  |  |  | |
|  | Excess Change in  Hispanic Pop. % | — | 0.056 |  | — | 0.039 |  | — | 0.042 | |
|  |  |  | (0.103) |  |  | (0.115) |  |  | (0.108) | |
|  | Excess Change in  Black Pop. % | — | -0.091 |  | — | -0.064 |  | — | -0.073 | |
|  |  |  | (0.136) |  |  | (0.136) |  |  | (0.130) | |
|  | Excess Change in  Unemployment | — | 0.243 |  | — | 0.388 |  | — | 0.371 | |
|  |  |  | (0.293) |  |  | (0.259) |  |  | (0.289) | |
|  |  |  |  |  |  |  |  |  |  | |
| ***(Table S4. Continued)*** | | | | | | | | | | |
| Quarter $\times$ Excess Unemployment | | | | | | | | | | |
| Pre-Pandemic Birth  and Conception | Apr–Jun 2018 $\times$  Unemp. | — | -0.232 |  | — | -0.296 |  | — | -0.298 | |
|  |  |  | (0.323) |  |  | (0.267) |  |  | (0.302) | |
|  | Jul–Sep 2018 $\times$  Unemp. | — | -0.668^†^ |  | — | -0.673* |  | — | -0.694^†^ | |
|  |  |  | (0.368) |  |  | (0.321) |  |  | (0.366) | |
|  | Oct–Dec 2018 $\times$  Unemp. | — | -0.327 |  | — | -0.375 |  | — | -0.539 | |
|  |  |  | (0.357) |  |  | (0.323) |  |  | (0.338) | |
|  | Jan–Mar 2019 $\times$  Unemp. | — | -0.235 |  | — | -0.337 |  | — | -0.349 | |
|  |  |  | (0.241) |  |  | (0.209) |  |  | (0.220) | |
|  | Apr–Jun 2019 $\times$  Unemp. | — | -0.193 |  | — | -0.313 |  | — | -0.367 | |
|  |  |  | (0.312) |  |  | (0.293) |  |  | (0.319) | |
|  | Jul–Sep 2019 $\times$  Unemp. | — | -0.793** |  | — | -0.879** |  | — | -0.887** | |
|  |  |  | (0.265) |  |  | (0.264) |  |  | (0.278) | |
|  | Oct–Dec 2019 $\times$  Unemp. | — | -0.065 |  | — | -0.150 |  | — | -0.200 | |
|  |  |  | (0.302) |  |  | (0.276) |  |  | (0.284) | |
| Pandemic Birth,  but Not Conception |  |  |  |  |  |  |  |  |  | |
|  | Jan–Mar 2020 $\times$  Unemp. | — | -0.253 |  | — | -0.345 |  | — | -0.371 | |
|  |  |  | (0.263) |  |  | (0.261) |  |  | (0.262) | |
|  | Apr–Jun 2020 $\times$  Unemp. | — | -0.106 |  | — | -0.235 |  | — | -0.215 | |
|  |  |  | (0.277) |  |  | (0.244) |  |  | (0.275) | |
|  | Jul–Sep 2020 $\times$  Unemp. | — | 0.118 |  | — | 0.001 |  | — | 0.023 | |
|  |  |  | (0.293) |  |  | (0.266) |  |  | (0.296) | |
| Pandemic Birth  and Conception |  |  |  |  |  |  |  |  |  | |
|  | Oct–Dec 2020 $\times$  Unemp. | — | 0.082 |  | — | -0.032 |  | — | -0.012 | |
|  |  |  | (0.284) |  |  | (0.242) |  |  | (0.276) | |
|  | Jan–Mar 2021 $\times$  Unemp. | — | -0.280 |  | — | -0.421^†^ |  | — | -0.388 | |
|  |  |  | (0.292) |  |  | (0.249) |  |  | (0.285) | |
|  | Apr–Jun 2021 $\times$  Unemp. | — | -0.389 |  | — | -0.513^†^ |  | — | -0.515^†^ | |
|  |  |  | (0.308) |  |  | (0.269) |  |  | (0.307) | |
|  | Jul–Sep 2021 $\times$  Unemp. | — | -0.280 |  | — | -0.495^†^ |  | — | -0.404 | |
|  |  |  | (0.329) |  |  | (0.260) |  |  | (0.319) | |
|  | Oct–Dec 2021 $\times$  Unemp. | — | -0.542 |  | — | -0.572^†^ |  | — | -0.660^†^ | |
|  |  |  | (0.337) |  |  | (0.300) |  |  | (0.342) | |
|  | Jan–Mar 2022 $\times$  Unemp. | — | -0.132 |  | — | -0.198 |  | — | -0.267 | |
|  |  |  | (0.318) |  |  | (0.285) |  |  | (0.301) | |
|  | Apr–Jun 2022 $\times$  Unemp. | — | -0.196 |  | — | -0.273 |  | — | -0.339 | |
|  |  |  | (0.313) |  |  | (0.296) |  |  | (0.304) | |
|  |  |  |  |  |  |  |  |  |  | |
|  | Constant | -0.505*** | -0.166 |  | -0.588*** | 0.045 |  | -0.551*** | 0.008 | |
|  |  | (0.089) | (0.504) |  | (0.078) | (0.478) |  | (0.142) | (0.485) | |
|  | R^2^ | 0.520 | 0.546 |  | 0.506 | 0.534 |  | 0.519 | 0.546 | |
| *Note*. *N* of counties = 55, *N* of observations = 990; Months are abbreviated; Pop. = population; Unemp. = excess unemployment rate; excess rates calculated based on differences from average for 2014–2017, lagged three quarters (see text for details); Numbers in parentheses are clustered standard errors; All models include county fixed effects. ^a:^ Compared to Jan–Mar 2018; ^b^ Trump majority denotes ≥ 50% 2020 Trump vote share. ^c:^ Trump supermajority denotes ≥ 60% 2020 Trump vote share. ^†^ *p*  < .10, * *p*  < .05, ** *p* < .01, *** *p* < .001 (two-tailed tests). | | | | | | | | | | |
| Table S5. Quarterly Change in Excess Fertility Rates by 2020 Trump Vote Share Before and During the COVID-19 Pandemic, Non-White Women | | | | | | | | | |  |
|  |  | Trump Majority ^b^ | |  | Trump Supermajority ^c^ | |  | Trump % Vote Share | |  |
|  | Birth Quarter ^a^ | Model 1A | Model 2A |  | Model 1B | Model 2B |  | Model 1C | Model 2C |  |
| Pre-Pandemic Birth  and Conception | Apr–Jun 2018 | -0.180 | 1.170 |  | -0.202 | 1.126 |  | -0.468 | 0.756 |  |
|  |  | (0.298) | (1.751) |  | (0.237) | (1.719) |  | (1.121) | (1.919) |  |
|  | Jul–Sep 2018 | -0.481* | -0.327 |  | -0.598** | -0.061 |  | 0.026 | 0.289 |  |
|  |  | (0.222) | (1.143) |  | (0.195) | (1.163) |  | (1.040) | (1.419) |  |
|  | Oct–Dec 2018 | -0.193 | 2.087 |  | -0.337^†^ | 2.216 |  | 0.765 | 2.566 |  |
|  |  | (0.230) | (1.399) |  | (0.196) | (1.395) |  | (1.016) | (1.679) |  |
|  | Jan–Mar 2019 | -0.420^†^ | 1.888 |  | -0.237 | 1.666 |  | -0.012 | 1.685 |  |
|  |  | (0.238) | (1.467) |  | (0.229) | (1.295) |  | (1.038) | (1.415) |  |
|  | Apr–Jun 2019 | -0.196 | -0.384 |  | -0.300^†^ | -0.153 |  | 0.300 | 0.212 |  |
|  |  | (0.176) | (1.083) |  | (0.169) | (1.078) |  | (0.917) | (1.283) |  |
|  | Jul–Sep 2019 | -0.064 | 0.526 |  | -0.209 | 0.481 |  | 0.234 | 0.714 |  |
|  |  | (0.347) | (1.655) |  | (0.297) | (1.718) |  | (1.510) | (2.209) |  |
|  | Oct–Dec 2019 | -0.285 | 1.298 |  | -0.312 | 1.239 |  | -0.190 | 1.097 |  |
|  |  | (0.286) | (1.394) |  | (0.226) | (1.378) |  | (1.110) | (1.679) |  |
| Pandemic Birth, but Not Conception |  |  |  |  |  |  |  |  |  |  |
|  | Jan–Mar 2020 | -0.576** | 1.038 |  | -0.518** | 1.106 |  | -0.018 | 1.269 |  |
|  |  | (0.210) | (1.385) |  | (0.188) | (1.361) |  | (1.005) | (1.572) |  |
|  | Apr–Jun 2020 | -0.804** | 0.317 |  | -0.853*** | 0.227 |  | -0.716 | 0.144 |  |
|  |  | (0.249) | (1.144) |  | (0.216) | (1.137) |  | (1.109) | (1.573) |  |
|  | Jul–Sep 2020 | -1.504*** | 0.010 |  | -1.473*** | -0.013 |  | -1.566 | -0.328 |  |
|  |  | (0.358) | (1.359) |  | (0.288) | (1.329) |  | (1.219) | (1.777) |  |
| Pandemic Birth  and Conception |  |  |  |  |  |  |  |  |  |  |
|  | Oct–Dec 2020 | -1.462*** | -0.099 |  | -1.319*** | -0.026 |  | -1.917^†^ | -0.862 |  |
|  |  | (0.326) | (1.201) |  | (0.269) | (1.149) |  | (1.041) | (1.430) |  |
|  | Jan–Mar 2021 | -1.720*** | -0.976 |  | -1.539*** | -0.742 |  | -1.135 | -0.989 |  |
|  |  | (0.301) | (1.186) |  | (0.263) | (1.193) |  | (0.921) | (1.514) |  |
|  | Apr–Jun 2021 | -0.794* | 0.072 |  | -0.797** | 0.108 |  | -0.172 | -0.282 |  |
|  |  | (0.305) | (1.223) |  | (0.233) | (1.162) |  | (1.205) | (1.942) |  |
|  | Jul–Sep 2021 | -0.747^†^ | 0.368 |  | -0.717* | 0.256 |  | -0.646 | 0.477 |  |
|  |  | (0.400) | (1.205) |  | (0.330) | (1.144) |  | (1.193) | (1.645) |  |
|  | Oct–Dec 2021 | -0.953** | 0.135 |  | -0.878** | 0.153 |  | 0.734 | 1.214 |  |
|  |  | (0.350) | (1.224) |  | (0.268) | (1.131) |  | (1.003) | (1.733) |  |
|  | Jan–Mar 2022 | -0.420 | 1.167 |  | -0.231 | 1.033 |  | -0.023 | 0.262 | |
|  |  | (0.298) | (1.187) |  | (0.265) | (1.104) |  | (0.936) | (1.387) | |
|  | Apr–Jun 2022 | -0.356 | 1.133 |  | -0.366 | 1.013 |  | 0.903 | 1.689 | |
|  |  | (0.302) | (1.251) |  | (0.251) | (1.111) |  | (1.016) | (1.404) | |
|  | | | | | | | | | | |
| ***(Table S5. Continued)*** | | | | | | | | | | |
| Quarter $\times$ Trump Support (i.e., Partisan Excess Fertility Gap) | | | | | | | | | | |
| Pre-Pandemic Birth  and Conception | Apr–Jun 2018 $\times$  Trump | -0.080 | 0.089 |  | -0.059 | 0.005 |  | 0.005 | 0.011 | |
|  |  | (0.459) | (0.463) |  | (0.701) | (0.667) |  | (0.022) | (0.021) | |
|  | Jul–Sep 2018 $\times$  Trump | -0.280 | -0.277 |  | 0.009 | -0.001 |  | -0.012 | -0.015 | |
|  |  | (0.389) | (0.416) |  | (0.608) | (0.633) |  | (0.021) | (0.023) | |
|  | Oct–Dec 2018 $\times$  Trump | -0.560 | -0.288 |  | -0.505 | -0.326 |  | -0.024 | -0.011 | |
|  |  | (0.389) | (0.347) |  | (0.659) | (0.632) |  | (0.021) | (0.021) | |
|  | Jan–Mar 2019 $\times$  Trump | -0.079 | 0.192 |  | -1.243* | -1.123* |  | -0.009 | 0.002 | |
|  |  | (0.463) | (0.498) |  | (0.559) | (0.544) |  | (0.022) | (0.023) | |
|  | Apr–Jun 2019 $\times$  Trump | -0.412 | -0.396 |  | -0.377 | -0.341 |  | -0.013 | -0.011 | |
|  |  | (0.358) | (0.368) |  | (0.575) | (0.571) |  | (0.019) | (0.019) | |
|  | Jul–Sep 2019 $\times$  Trump | -0.549 | -0.414 |  | -0.475 | -0.376 |  | -0.010 | -0.004 | |
|  |  | (0.611) | (0.616) |  | (0.969) | (0.958) |  | (0.031) | (0.030) | |
|  | Oct–Dec 2019 $\times$  Trump | -0.285 | -0.114 |  | -0.525 | -0.419 |  | -0.004 | 0.005 | |
|  |  | (0.475) | (0.471) |  | (0.790) | (0.797) |  | (0.023) | (0.023) | |
| Pandemic Birth,  but Not Conception |  |  |  |  |  |  |  |  |  | |
|  | Jan–Mar 2020 $\times$  Trump | -0.187 | -0.016 |  | -0.780 | -0.676 |  | -0.013 | -0.004 | |
|  |  | (0.435) | (0.444) |  | (0.734) | (0.725) |  | (0.021) | (0.022) | |
|  | Apr–Jun 2020 $\times$  Trump | -0.187 | -0.022 |  | -0.167 | -0.049 |  | -0.003 | 0.005 | |
|  |  | (0.451) | (0.445) |  | (0.712) | (0.710) |  | (0.023) | (0.022) | |
|  | Jul–Sep 2020 $\times$  Trump | -0.063 | 0.096 |  | -0.329 | -0.219 |  | 0.001 | 0.009 | |
|  |  | (0.512) | (0.511) |  | (0.657) | (0.646) |  | (0.024) | (0.023) | |
| Pandemic Birth  and Conception |  |  |  |  |  |  |  |  |  | |
|  | Oct–Dec 2020 $\times$  Trump | 0.118 | 0.226 |  | -0.536 | -0.442 |  | 0.010 | 0.018 | |
|  |  | (0.454) | (0.458) |  | (0.515) | (0.507) |  | (0.020) | (0.021) | |
|  | Jan–Mar 2021 $\times$  Trump | -0.012 | 0.216 |  | -1.062^†^ | -0.844 |  | -0.012 | 0.003 | |
|  |  | (0.462) | (0.468) |  | (0.535) | (0.567) |  | (0.018) | (0.021) | |
|  | Apr–Jun 2021 $\times$  Trump | -0.462 | -0.114 |  | -1.088 | -0.846 |  | -0.016 | 0.005 | |
|  |  | (0.487) | (0.560) |  | (0.786) | (0.846) |  | (0.024) | (0.029) | |
|  | Jul–Sep 2021 $\times$  Trump | -0.057 | -0.078 |  | -0.301 | -0.299 |  | -0.002 | -0.003 | |
|  |  | (0.570) | (0.696) |  | (0.639) | (0.719) |  | (0.023) | (0.027) | |
|  | Oct–Dec 2021 $\times$  Trump | -0.329 | 0.344 |  | -1.209^†^ | -0.827 |  | -0.036^†^ | -0.022 | |
|  |  | (0.487) | (0.681) |  | (0.652) | (0.705) |  | (0.019) | (0.027) | |
|  | Jan–Mar 2022 $\times$  Trump | 0.272 | 0.989 |  | -0.416 | -0.016 |  | -0.006 | 0.020 | |
|  |  | (0.514) | (0.702) |  | (0.669) | (0.740) |  | (0.020) | (0.027) | |
|  | Apr–Jun 2022 $\times$  Trump | -0.390 | -0.102 |  | -0.876 | -0.585 |  | -0.028 | -0.017 | |
|  |  | (0.456) | (0.631) |  | (0.605) | (0.635) |  | (0.020) | (0.026) | |
|  |  |  |  |  |  |  |  |  |  | |
|  | Excess Change in  Hispanic Pop. % | — | 0.134 |  | — | 0.138 |  | — | 0.207^†^ | |
|  |  |  | (0.118) |  |  | (0.148) |  |  | (0.120) | |
|  | Excess Change in  Black Pop. % | — | 0.038 |  | — | 0.045 |  | — | 0.013 | |
|  |  |  | (0.243) |  |  | (0.244) |  |  | (0.247) | |
|  | Excess Change in  Unemployment | — | -0.728 |  | — | -0.652 |  | — | -0.788 | |
|  |  |  | (0.568) |  |  | (0.516) |  |  | (0.546) | |
|  |  |  |  |  |  |  |  |  |  | |
| ***(Table S5. Continued)*** | | | | | | | | | | |
| Quarter $\times$ Excess Unemployment | | | | | | | | | | |
| Pre-Pandemic Birth  and Conception | Apr–Jun 2018 $\times$  Unemp. | — | 0.745 |  | — | 0.703 |  | — | 0.808 | |
|  |  |  | (0.952) |  |  | (0.895) |  |  | (0.930) | |
|  | Jul–Sep 2018 $\times$  Unemp. | — | 0.030 |  | — | 0.251 |  | — | 0.022 | |
|  |  |  | (0.625) |  |  | (0.620) |  |  | (0.614) | |
|  | Oct–Dec 2018 $\times$  Unemp. | — | 1.385^†^ |  | — | 1.516* |  | — | 1.424^†^ | |
|  |  |  | (0.773) |  |  | (0.756) |  |  | (0.759) | |
|  | Jan–Mar 2019 $\times$  Unemp. | — | 1.173 |  | — | 0.957 |  | — | 1.124^†^ | |
|  |  |  | (0.717) |  |  | (0.599) |  |  | (0.664) | |
|  | Apr–Jun 2019 $\times$  Unemp. | — | 0.148 |  | — | 0.259 |  | — | 0.253 | |
|  |  |  | (0.533) |  |  | (0.508) |  |  | (0.501) | |
|  | Jul–Sep 2019 $\times$  Unemp. | — | 0.436 |  | — | 0.451 |  | — | 0.533 | |
|  |  |  | (0.779) |  |  | (0.766) |  |  | (0.775) | |
|  | Oct–Dec 2019 $\times$  Unemp. | — | 0.919 |  | — | 0.875 |  | — | 0.990 | |
|  |  |  | (0.705) |  |  | (0.662) |  |  | (0.675) | |
| Pandemic Birth,  but Not Conception |  |  |  |  |  |  |  |  |  | |
|  | Jan–Mar 2020 $\times$  Unemp. | — | 0.873 |  | — | 0.834 |  | — | 0.925 | |
|  |  |  | (0.634) |  |  | (0.597) |  |  | (0.613) | |
|  | Apr–Jun 2020 $\times$  Unemp. | — | 0.701 |  | — | 0.646 |  | — | 0.770 | |
|  |  |  | (0.560) |  |  | (0.529) |  |  | (0.542) | |
|  | Jul–Sep 2020 $\times$  Unemp. | — | 0.845 |  | — | 0.787 |  | — | 0.921 | |
|  |  |  | (0.618) |  |  | (0.577) |  |  | (0.600) | |
| Pandemic Birth  and Conception |  |  |  |  |  |  |  |  |  | |
|  | Oct–Dec 2020 $\times$  Unemp. | — | 0.850 |  | — | 0.805 |  | — | 0.931 | |
|  |  |  | (0.580) |  |  | (0.532) |  |  | (0.560) | |
|  | Jan–Mar 2021 $\times$  Unemp. | — | 0.779 |  | — | 0.683 |  | — | 0.834 | |
|  |  |  | (0.570) |  |  | (0.518) |  |  | (0.549) | |
|  | Apr–Jun 2021 $\times$  Unemp. | — | 0.797 |  | — | 0.690 |  | — | 0.881 | |
|  |  |  | (0.582) |  |  | (0.520) |  |  | (0.559) | |
|  | Jul–Sep 2021 $\times$  Unemp. | — | 0.622 |  | — | 0.568 |  | — | 0.674 | |
|  |  |  | (0.572) |  |  | (0.518) |  |  | (0.550) | |
|  | Oct–Dec 2021 $\times$  Unemp. | — | 1.190^†^ |  | — | 0.864 |  | — | 0.911 | |
|  |  |  | (0.604) |  |  | (0.529) |  |  | (0.593) | |
|  | Jan–Mar 2022 $\times$  Unemp. | — | 1.538* |  | — | 1.024^†^ |  | — | 1.286* | |
|  |  |  | (0.657) |  |  | (0.512) |  |  | (0.638) | |
|  | Apr–Jun 2022 $\times$  Unemp. | — | 1.000 |  | — | 0.909 |  | — | 0.900 | |
|  |  |  | (0.685) |  |  | (0.547) |  |  | (0.658) | |
|  |  |  |  |  |  |  |  |  |  | |
|  | Constant | -0.675** | -1.982^†^ |  | -0.691*** | -1.956^†^ |  | -0.736** | -2.136* | |
|  |  | (0.221) | (1.071) |  | (0.175) | (1.035) |  | (0.274) | (1.065) | |
|  | R^2^ | 0.317 | 0.330 |  | 0.322 | 0.329 |  | 0.318 | 0.327 | |
| *Note*. *N* of counties = 56, *N* of observations = 1,008; Months are abbreviated; Pop. = population; Unemp. = excess unemployment rate; excess rates calculated based on differences from average for 2014–2017, lagged three quarters (see text for details); Numbers in parentheses are clustered standard errors; All models include county fixed effects. ^a:^ Compared to Jan–Mar 2018; ^b^ Trump majority denotes ≥ 50% 2020 Trump vote share. ^c:^ Trump supermajority denotes ≥ 60% 2020 Trump vote share. ^†^ *p*  < .10, * *p*  < .05, ** *p* < .01, *** *p* < .001 (two-tailed tests). | | | | | | | | | | |

**Figure S1. Quarterly Excess Fertility by 2020 County Partisanship, Controlling for Education**

Panel A. Trump Majority ^a^

**** Excess Fertility Republican-Leaning – Democratic Leaning

Difference

Panel B. Trump Supermajority ^b^

Excess Fertility Republican-Leaning – Democratic Leaning

Difference

Panel C. Percentage Trump Support

Excess Fertility Excess Fertility Change per

1% Trump Increase

*Note*. Excess fertility is calculated as the fertility rate for a quarter (e.g., April-June 2018) - the same season’s (e.g., April–June) 2014–2017 average. Models control for county race/ethnicity, unemployment changes, percent with less than a high school education, percent with a high school education, percent with a B.A. or more interacted with quarter, and include time and county fixed effects. *N* = 56 counties, *N* = 1,008 observations. Partisan gap figures show the Republican-leaning area - Democrat-leaning area difference in excess fertility and 95% confidence intervals for that difference. The light purple graph area shading shows pandemic births and pre-pandemic conceptions. The dark purple graph area shading shows pandemic births and conceptions. ^a:^ Trump majority denotes ≥50% Trump 2020 vote share. ^b:^ Trump Supermajority denotes ≥ 60% 2020 Trump vote share.

**Figure S2. SARIMA Model, Deviation of Actual Fertility from Forecasted for All Counties**

**
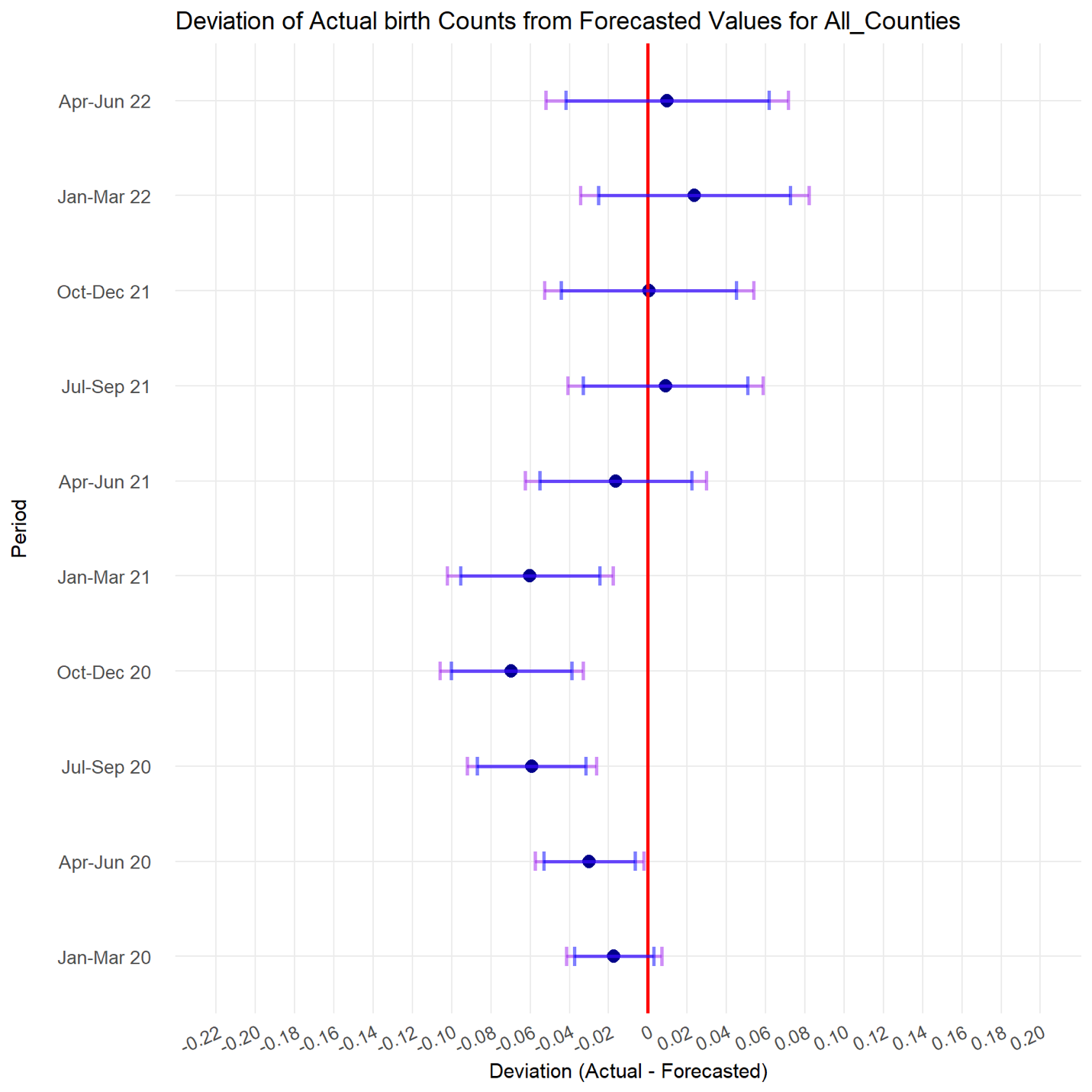
**

*Note*. SARIMA is seasonally adjusted autoregressive integrated moving average models. Deviations from forecasts based on trends from 2014—2019. 95% (90%) CIs are shown in purple (blue); if the CIs do not include 0, the red line, then quarter fertility significantly deviated from the forecasted trend at the .05 (.10) level. *N* = 56 counties, *N* = 1,008 observations. The light purple graph area shading shows pandemic births and pre-pandemic conceptions. The dark purple graph area shading shows pandemic births and conceptions.

**Figure S3. SARIMA Model, Deviation of Actual Fertility from Forecasted by 2020 County Partisanship**

Minority Trump Vote (<50%) Majority Trump Vote ($\geq$50%)

**
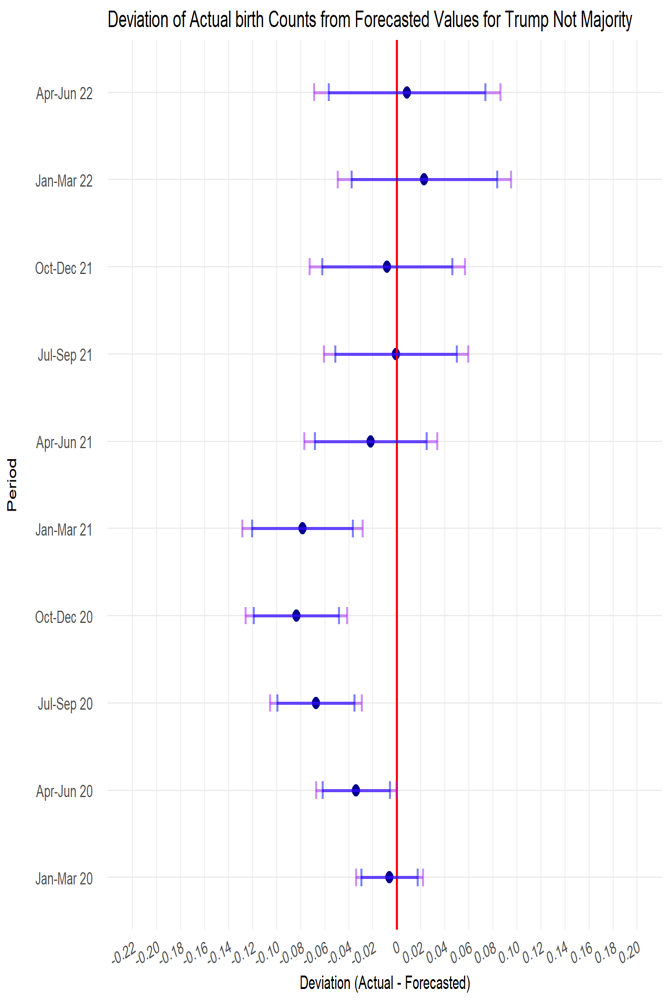
**
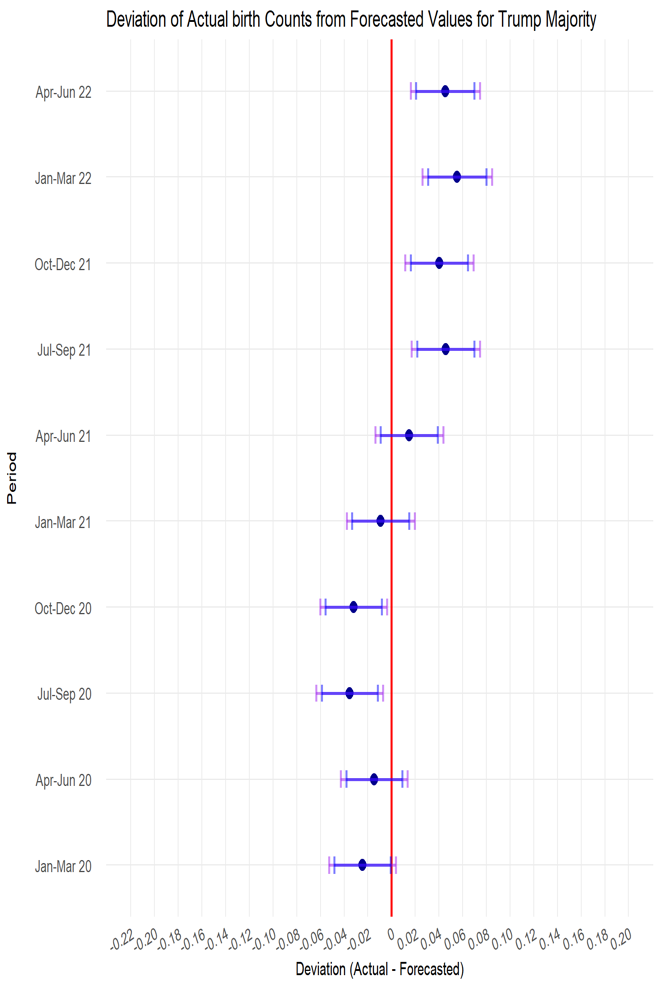


*Note.* SARIMA is seasonally adjusted autoregressive integrated moving average models. Models were computed separately for counties in which Trump did not receive a majority of 2020 votes, <50% (left panel), and those in which Trump received a majority of 2020 votes, $\geq$50%, (right panel). Deviations from forecasts based on trends from 2014–2019. 95% (90%) CIs are shown in purple (blue); if the CIs do not include 0, the red line, then fertility significantly deviated from the forecasted trend at the .05 (.10) level. *N* = 56 counties, *N* = 1,008 observations. The light purple graph area shading shows pandemic births and pre-pandemic conceptions. The dark purple graph area shading shows pandemic births and conceptions.

**Figure S4. Percent Change in Quarterly Fertility Relative to Same Quarter Prior Year by 2020 County Partisanship**

Panel A. Trump Majority ^a^

% Change in Fertility Republican-Leaning – Democratic Leaning

Difference

Panel B. Trump Supermajority ^b^

% Change in Fertility Republican-Leaning – Democratic Leaning

Difference

Panel C. Percentage Trump Support

% Change in Fertility % Change in Fertility per

1% Trump Increase

*Note.* Percent change in fertility rate relative to same quarter the prior year, e.g., January–March 2021 vs. January-March 2020. Models control for county race/ethnicity and unemployment changes, and include time and county fixed effects. *N* = 56 counties. *N* = 1,008 observations. Within each panel, the left image shows estimated percent change in fertility rate for Republican-leaning and Democratic-leaning counties; the right image shows the magnitude of the partisan gap and 95% CIs, when the CIs do not include 0 the partisan gap is significant at the .05 level. The light purple graph area shading shows pandemic births and pre-pandemic conceptions. The dark purple graph area shading shows pandemic births and conceptions. ^a:^ Trump majority denotes ≥50% 2020 Trump vote share. ^b:^ Trump Supermajority denotes ≥60% 2020 Trump vote share.
